# The N‐terminal fragment of histone deacetylase 4 (1‐669aa) promotes chondrocyte apoptosis via the p53‐dependent endoplasmic reticulum stress pathway

**DOI:** 10.1111/jcmm.70135

**Published:** 2024-10-20

**Authors:** Li Guo, Xuhao Zhuo, Chengyang Lu, Hua Guo, Zhi Chen, Gaige Wu, Fengrui Liu, Xiaochun Wei, Xueqin Rong, Pengcui Li

**Affiliations:** ^1^ Department of Orthopedics, Shanxi Key Laboratory of Bone and Soft Tissue Injury Repair Second Hospital of Shanxi Medical University Taiyuan Shanxi China; ^2^ Department of Orthopedics People's Hospital of Xinzhou Xinzhou Shanxi China; ^3^ Department of Pain Medicine Center Central Hospital of Sanya Sanya Hainan China

**Keywords:** apoptosis, chondrocytes, endoplasmic reticulum stress, histone deacetylase 4, p53

## Abstract

Exogenous administration of the histone deacetylation 4 (HDAC4) protein can effectively delay osteoarthritis (OA) progression. However, HDAC4 is unstable and easily degrades into N‐terminal (HDAC4‐NT) and C‐terminal fragments, and the HDAC4‐NT can exert biological effects, but little is known about its role in chondrocytes and cartilage. Thus, the roles of HDAC4‐NT fragments (1‐289aa, 1‐326aa and 1‐669aa) in chondrocytes and cartilage were evaluated via real‐time cell analysis (RTCA), safranin O staining, Sirius Red staining and nanoindentation. Molecular mechanisms were profiled via whole‐transcriptome sequencing (RNA‐seq) and verified in vitro and in vivo by a live cell real‐time monitoring system, flow cytometry, western blotting and immunohistochemistry. The results showed that 1‐669aa induced chondrocyte death and cartilage injury significantly, and the differentially expressed genes (DEGs) were enriched mainly in the apoptotic term and p53 signalling pathway. The validation experiments showed that 1‐669aa induced chondrocyte apoptosis via the endoplasmic reticulum stress (ERS) pathway, and up‐regulated p53 expression was essential for this process. Thus, we concluded that the HDAC4‐NT fragment 1‐669aa induces chondrocyte apoptosis via the p53‐dependent ERS pathway, suggesting that in addition to overexpressing HDAC4, preventing it from degradation may be a new strategy for the treatment of OA.

## 1 INTRODUCTION

Osteoarthritis (OA) is a common chronic orthopaedic disease. It can not only lead to disability but negatively impact people's physical and mental well‐being, giving rise to an enormous burden for health and social care systems globally.^1^ However, the clinical therapy of OA is still limited to symptomatic treatments, such as pain relief and swelling reduction, and joint replacement at more advanced disease stages. The question remains on how to address the progression of OA efficiently.^2^

Histone deacetylase 4 (HDAC4) is a class II histone deacetylase (HDACs) that is mainly distributed in brain, muscle, and cartilage.^3^, ^4^ Studies have shown that HDAC4 controls chondrocyte hypertrophy and endochondral bone formation by inhibiting the function of myocyte‐specific enhancer factor 2C (MEF2C) and runt‐related transcription factor 2 (Runx‐2).^5^, ^6^, ^7^ Additionally, our previous studies and those of others revealed that decreased HDAC4 expression in cartilage accelerates the pathogenesis of osteoarthritis (OA),^8^, ^9^ and upregulating HDAC4 expression might become a new treatment method for OA.^10^, ^11^

**FIGURE 1:**
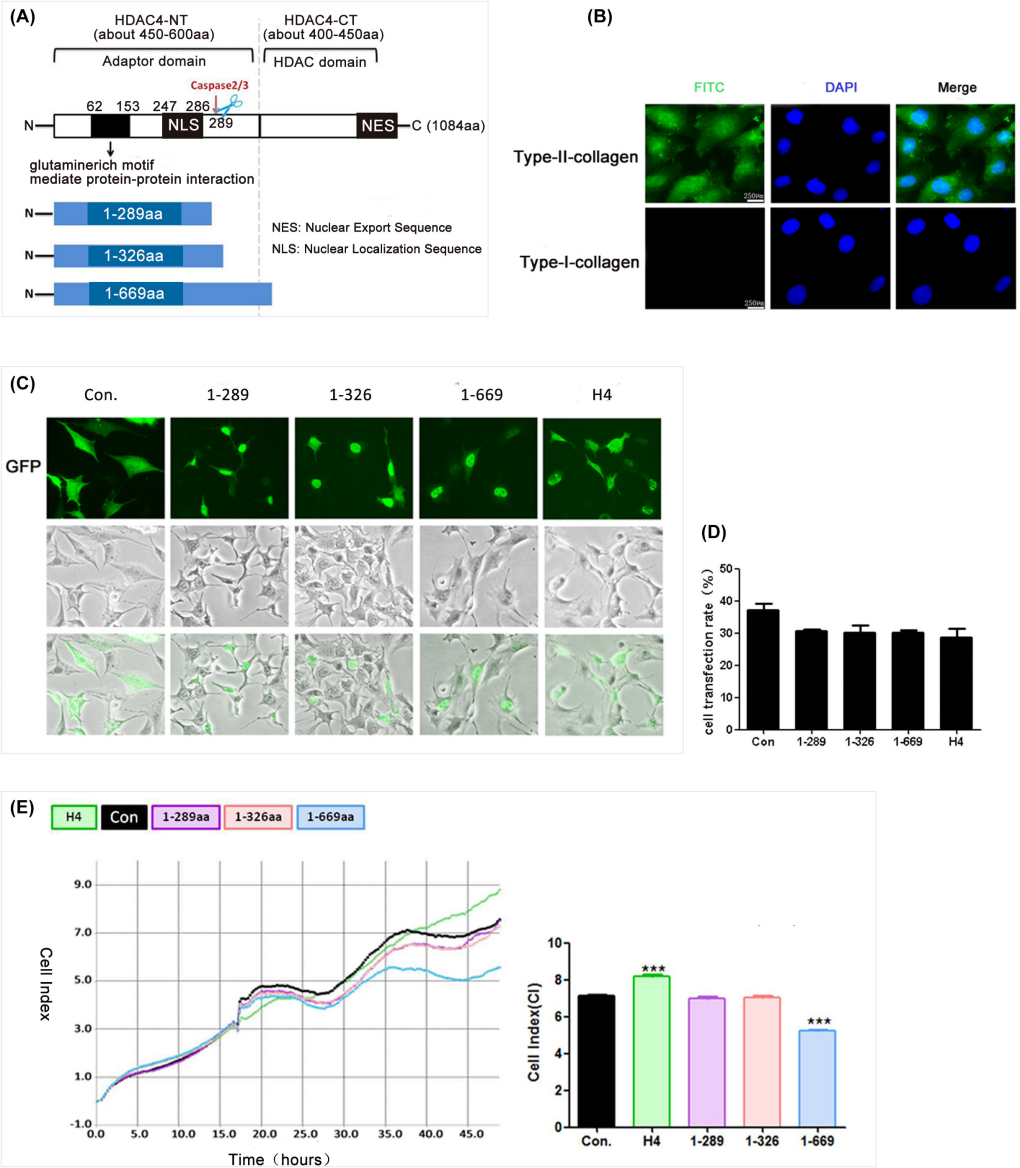
The HDAC4‐NT fragment (1‐669aa) induced chondrocytes death. (A) A schematic diagram of HDAC4 protein and HDAC4‐NT fragment. (B) Immunofluorescence assay results showed that cells were positive for type II collagen (green) and negative for type I collagen (green, scale bar: 250 μm). (C) The transfection efficiency in each group was observed by fluorescence microscopy. The EP, HDAC4‐NT fragments and full‐length HDAC4 carried GFP; green; scale bar: 50 μm. (D) The percentage of GFP‐positive cells in all cells with green fluorescence were quantified (*n* = 3). (E) Representative cell survival rates of groups were detected by RTCA assay, the data were quantified by average cell index (CI), ***p < 0.001. (*n* = 3).

However, the HDAC4 protein is easily degraded, with a half‐life of less than 8 h, and the N‐terminal fragment of HDAC4 (HDAC4‐NT) can accumulate in cell nuclei where it exerts biological effects independently of the HDAC domain.^12^ For example, HDAC4‐NT can cause cardiomyocyte hypertrophy^13^ or protect cardiac function^14^, ^15^; the 1‐289aa fragment can trigger human osteosarcoma cell (U2OS cell) death^16^, ^17^; and a short fragment of the HDAC4‐NT preserves photoreceptors and restores visual function in the retinitis pigmentosa.^18^ However, little is known about the role of HDAC4‐NT in chondrocytes and cartilage. Therefore, in this study, we explored the function of HDAC4‐NT fragments (1‐289aa, 1‐326aa and 1‐669aa) in chondrocytes and cartilage via in vitro and in vivo experiments, and the underlying molecular mechanisms were investigated via RNA‐seq. Among these three fragments, the HDAC4‐N terminal fragment 1‐289aa is generated by caspase 2‐ and caspase 3‐mediated cleavage of HDAC4 at Asp289.^12^, ^16^, ^17^ 1‐669aa includes almost all the important binding sites and posttranslational modifications (PTMs) sites in the HDAC4‐NT fragment.^4^, ^19^, ^20^, ^21^, ^22^, ^23^, ^24^, ^25^, ^26^ 1‐326aa includes the Asp289 site, and it is used to further determine the function of 1‐289aa (Figure 1A). Understanding the role and molecular mechanisms of HDAC4‐NT in chondrocytes and cartilage tissues can help us know how to use HDAC4 more effectively in the treatment of OA.

## 2 MATERIALS AND METHODS

### 2.1 Chondrocyte isolation and primary culture

Cartilage slices were removed from “relatively normal” cartilage samples (Mankin scores: 0–2) of the tibia that were obtained during total knee arthroplasty and washed in Dulbecco's modified Eagle's medium (DMEM) (Invitrogen, CA). Chondrocytes were isolated and cultured as previously described.^27^ After 48 h, immunocytochemical analysis of the chondrocyte phenotype was performed using anti‐type I collagen (ab34710; Abcam, USA) and anti‐type II collagen antibodies (ab34712; Abcam, USA).^28^ The cells were positive for type II collagen and negative for type I collagen (Figure 1B).

### 2.2 Overexpressing the HDAC4‐NT fragments 1‐289aa, 1‐326aa and 1‐669aa in chondrocytes

An empty plasmid (EP), plasmids carrying 1‐289aa, 1‐326aa and 1‐669aa and full‐length HDAC4 (H4) were constructed by GeneChem (CHN). Human chondrocytes were plated and transfected at a confluence of 70%–90%. Plasmid DNAs were prepared and transfected into cells using Lipofectamine 3000 (Lipo3000, Invitrogen, CA, USA) according to the manufacturer's protocol. After forty‐eight hours, the transfection efficiency was determined via fluorescence imaging (Olympus, JPN) and flow cytometry (CyFlow Cube 6; Partec, Germany). Cell survival was monitored using real‐time cell analysis (RTCA) and a live‐cell imaging system (Leica, GER), and the total RNA was isolated from the chondrocytes for RNA‐seq analysis.

### 2.3 Real‐time cell analysis (RTCA)

2 × 10^3^–4 × 10^3^ cells were added to each well of a 96‐well plate (ACEA Biosciences, Inc., USA) in medium supplemented with 10% FBS. The plate was incubated for a minimum of 30 min in a humidified (37°C), 5% CO_2_ incubator and then inserted into a real‐time cell electronic sensing system (ACEA Biosciences, Inc., USA); cell survival was monitored for 48 h.

### 2.4 Cell apoptosis measurement by flow cytometry

Cell viability was analysed by PE Annexin V and 7‐AAD staining kits (BD Pharmingen™, USA) according to the manufacturer's instructions. The percentages of live and dead cells were quantified by fluorescence‐activated cell sorting analysis on a MACS Quant instrument (Miltenyi Biotec GmbH, Bergisch Gladbach, GER). The experiment was repeated three times.

### 2.5 Western blotting

The cells were randomly divided into four groups: the empty adenovirus, 1‐669aa, HDAC4 and 1‐669aa + pifithrin‐α (PFT‐α, 506,132; Sigma–Aldrich, USA) groups. PFT‐α is a p53 inhibitor, and cells were pretreated with 5 μM PFT‐α for 0.5 h followed by treatment with 1‐669aa adenovirus for 48 h.^29^ After treatment, total protein was isolated from the chondrocytes and quantified by a Bradford Protein Assay Reagent Kit (Bio‐Rad, USA). Twenty micrograms of total protein were separated via 10% SDS–PAGE under reducing conditions. The proteins were then transferred to an NC membrane (Bio‐Rad, USA) and probed with polyclonal antibodies against Caspase 3 (sc‐7272; Santa Cruz, USA), Caspase‐9 (sc‐56,076; Santa Cruz, USA), Caspase‐12 (sc‐21,747; Santa Cruz, USA), p53 (A0263; ABclonal, CHN), Bcl‐2 (A0208; ABclonal, CHN) and GAPDH (ab8245 Abcam, UK). The secondary antibodies were HRP‐conjugated goat anti‐mouse (AS003, ABclonal, CHN) or HRP‐conjugated goat anti‐rabbit (AS014, ABclonal, CHN) and were diluted at a ratio of 1:5000 in PBS‐T. The immunoreactive protein bands were visualized using Odyssey® CLx, and band densities were quantified using Empiria Studio Analysis Software.

### 2.6 RNA‐seq analysis of chondrocytes

Total RNA was extracted from chondrocytes from the EP, 1‐669aa, and full‐length HDAC4 DNA plasmid transfection groups 48 h after transfection using TRIzol reagent (Invitrogen, CA). RNA degradation and contamination were evaluated on 1% agarose gels. RNA purity was assessed using a NanoPhotometer spectrophotometer (IMPLEN, USA), RNA concentration was measured using a Qubit RNA Assay Kit in a Qubit 2.0 Fluorometer (Life Technologies, USA), and RNA integrity was assessed using the RNA Nano 6000 Assay Kit of the Bioanalyzer 2100 system (Agilent Technologies, USA).^30^

The sequencing library was constructed using the New England Biolabs (NEB) Next UltraTM RNA Library Prep Kit for Illumina according to the manufacturer's instructions (NEB, USA). The index of the reference genome was built using HISAT2 v2.0.5. Differential gene expression analysis between groups was performed using the DESeq2 R package v. 1.22.1 (USA). All the statistical analyses were conducted using the R statistical programming language. Genes with an adjusted *p* value of <0.05 according to DESeq2 were considered to be differentially expressed. Differentially expressed genes (DEGs) were defined as those with a fold change ≥2 and a *p* value ≤0.05. Gene Ontology (GO) enrichment analysis and Kyoto Encyclopaedia of Genes and Genomes (KEGG) pathway analysis were performed using the clusterProfiler R package 4.0 (CHN).^30^

### 2.7 Animals

Two‐month‐old male SD rats (180–230 g) were housed at the Experimental Animal Centre of Shanxi Medical University. All the rats were housed under specific pathogen‐free conditions in groups of 3 or 4 per individually ventilated cage. The rats were maintained under a 12‐h light–dark cycle at 21°C and were given a standard rodent diet and water. The animal protocols and surgical procedures that were used in this study were approved by the Institutional Review Board of the Second Hospital of Shanxi Medical University (Taiyuan, China; CMTT#: 2021014, Approval 2021).

### 2.8 In vivo experimental design

Healthy adult male SD rats (*n* = 60) were randomly divided into six groups: the normal control group, 1‐289aa adenoviral DNA treatment group, 1‐669aa group, sham group, anterior cruciate ligament transaction (ACLT) + empty adenoviral DNA (Ep.‐ Ad.) group and ACLT+1‐669aa group (*n* = 10 per group). On day 0, ACLT was surgically in the right knees of SD rats with a mean weight of 200 g. Intra‐articular injections of adenovirus DNA vectors (1 × 10^9^ PFU/mL, 40 μL) or saline (sham) were administered every 3 weeks beginning at 3 days after surgery, and the animals were euthanized at 8 weeks after treatment.^31^ The time points for injection were chosen based on previous research.^32^ The injection dose was chosen based on the results of the preliminary experiments (Figure S1). The degree of cartilage injury was evaluated using safranin O staining, Sirius Red staining and nanoindentation, and the protein expression of caspase‐3, caspase‐12, p53 and Bcl‐2 was measured via immunohistochemical staining. The ARRIVE‐Checklist for the design and execution of protocols for animal research and treatment was used.

### 2.9 Intra‐articular injection

Adenoviral DNA vectors or empty adenoviral vectors suspended in sterile 0.9% saline at a concentration of 1 × 10^9^ PFU/mL were intra‐articularly injected into the normal or operated knees. The concentration was chosen according to previous rodent studies.^32^ Rats were anaesthetised by an intraperitoneal injection of sodium pentobarbital (30 mg/kg).^33^ Intra‐articular injection was performed via the 2‐person technique with one holder and one injector, and the knee was fully extended and held in place by surgical forceps.

### 2.10 Tissue processing

All the animals were euthanized at 8 weeks after treatment. Rat cartilage explants were fixed in 10% formalin (Sigma–Aldrich, USA) for 72 h. The specimens were decalcified in Richman‐Gelfand‐Hill solution and processed in a Tissue‐Tek VIP 1000 tissue processor (Miles, USA). The femurs and tibiae of SD rats were hemisected in the midsagittal plane, and each half was embedded in a single block of Paraplast X‐tra (Thermo Fisher, USA). The blocks were trimmed to expose the tissue using a rotary microtome (Leica Microsystems Ltd., GER). Ten adjacent sections were collected at intervals of 0, 100 and 200 μm. Two serial 6‐μm‐thick sections from each interval were stained with safranin O.

### 2.11 Nanoindentation

This study determined the microelasticity of rat cartilage samples using a nanoindentation instrument (Piuma Nanoindenter, Optics11, Amsterdam, the Netherlands). A probe with a 5.17 N/m spring constant and 25 μm spherical indentation tip was used. During indentation, the spherical tip was brought into contact with the sample surface; load–indentation and load–time data were recorded. The probe displacement and probe displacement velocities were set to 10 μm and 18 μm/s, respectively. Young's modulus was derived from the load–indentation curves using the Hertz model. This model was applied to the loading dataset corresponding to 80% of the maximum load points; five unique points were measured per sample.

### 2.12 Immunohistochemistry

The immunohistochemistry detection was performed using a 3,3′‐diaminobenzidine (DAB) Histostain streptavidin peroxidase (SP) kit (Novex, Life Technologies, USA). The sections (6 μm) were digested with 5 mg/mL hyaluronidase in phosphate‐buffered saline (PBS) at 37°C for 10 min. Endogenous peroxidase was blocked by treating the sections with 3% hydrogen peroxide in methanol (Sigma–Aldrich, USA) at room temperature for 10 min. Nonspecific protein binding was blocked by incubation with a serum blocking solution (LI‐COR, GER) at room temperature for 10 min. The sections were incubated with specific antibodies at 4°C overnight. Thereafter, the sections were sequentially treated with biotinylated secondary antibody and SP conjugates at 37°C for 10 min and then developed in DAB chromogen (Invitrogen, CA) for 3 min. The sections were counterstained with haematoxylin (Invitrogen, CA) for 1 min. Photomicrographs were obtained with a Nikon E800 microscope (Nikon, USA).

### 2.13 Sirius red staining

The slides were incubated with Sirius Red solution for 1 h. After washing with PBS to remove the residual staining solution from the surface, the nuclei were stained with a haematoxylin staining solution (Mayer type) for 3–5 min; afterward, the slides were dehydrated frequently. Images were captured using a polarizing microscope. Type I collagen fibres were intense orange–yellow or bright red in colour, while type III collagen fibres were green.

### 2.14 Statistical analysis

SPSS 18.0 software (IBM, USA) was utilized for statistical analysis. Three replicates were performed for experiments in this study, and the data are shown as the mean ± standard deviation (SD). Differences between two groups and multiple groups were analysed using Student's *t*‐test and one‐way ANOVA, respectively. *p* < 0.05 was considered significant.

## 3 RESULTS

### 3.1 The HDAC4‐NT fragment 1‐669aa induced chondrocyte death

To explore the role of HDAC4‐NT in chondrocytes, plasmids carrying EP, 1‐289aa, 1‐326aa, 1‐669aa and H4 were separately transfected into human chondrocytes. The subcellular localization, transfection efficiency and cell survival were analysed 48 h after transfection. The results showed that in contrast to H4, which was mainly distributed in the cytoplasm, all three HDAC4‐NT fragments were distributed in the nucleus (Figure 1C), and the transfection efficiency of each group was approximately 30%–40% (Figure 1D, Figure S2). Interestingly, among the three HDAC4‐NT fragments, neither the 1‐289aa fragment nor the 1‐326aa fragment had a significant effect on cell survival, but the 1‐669aa fragment significantly decreased the survival of chondrocytes (Figure 1E). In addition, full‐length H4 effectively increased the survival of chondrocytes, which is consistent with our previous research results.^34^ The results indicated that 1‐669aa might induce chondrocyte death.

### 3.2 Intra‐articular injection of the HDAC4‐NT fragment 1‐669aa induced articular cartilage injury

**FIGURE 2:**
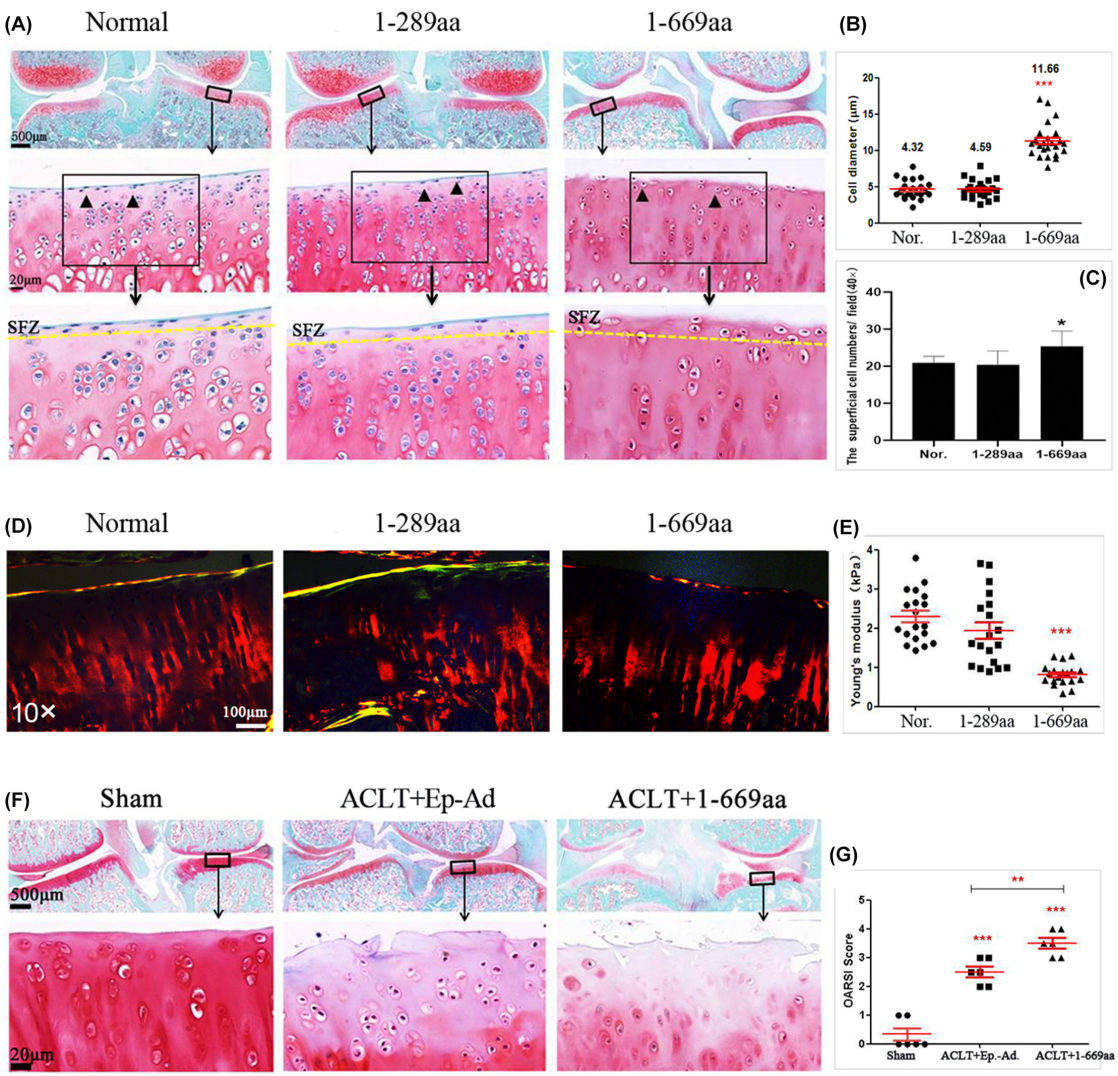
Intra‐articular HDAC4‐NT fragments (1‐669aa) injection induced the articular cartilage injury. (A) Safranin O and fast green staining results of normal, 1‐289aa and 1‐669aa treatment groups showed the degenerative degree of cartilage tissue, the figures in the first line (2×; scale bar: 500 μm), the figures in the second line (40×; scale bar: 20 μm), SFZ: The superficial zone. (B) the data were quantified by average diameter of the chondrocytes in the superficial zone, *p* < 0.001. (*n* = 24). (C) the data were quantified by average number of the chondrocytes in the superficial zone, *p* < 0.05 (*n*‐6). (D) Representative Sirius Red staining results. (10×; scale bar: 100 μm). (E) Average Young's modulus of groups was quantified (*n* = 6). (F) Representative Safranin O and fast green staining results of sham, ACLT+Ep.‐Ad. and ACLT+1‐669aa treatment groups, the figures in the first line (2×; scale bar: 500 μm), the figures in the second line (40×; scale bar: 20 μm). (G) the data were quantified by average OARIS scores of each group, **p < 0.01, ***p < 0.001. (*n* = 6).

To further verify the function of HDAC4‐NT, 1‐289aa and 1‐669aa adenoviral DNA vectors were intra‐articularly injected into the knee joint space of normal SD rats, and the degree of cartilage injury was evaluated at 8 weeks after treatment. The results of safranin O and fast green staining revealed that the integrity of the superficial zone of the cartilage was compromised, chondrocytes were enlarged and clustered, and chondrocyte numbers were decreased in the cartilage of the 1‐669aa group (Figure 2A–C). Especially in the superficial zone, the cell morphology changed from flat to round or oval, and the arrangement changed from parallel to the joint surface to clusters in the 1‐669aa group (Figure 2A); the cell diameter was significantly increased (Figure 2B), and the cell number was significantly increased too (Figure 2C). Sirius Red staining showed that in the 1‐669aa group, the collagen content of the superficial zone was significantly decreased, and discontinuity was observed (Figure 2D). Nanoindentation analysis also revealed that the 1‐669aa significantly decreased the mechanical properties of cartilage (Figure 2E). The above results showed that 1‐669aa can lead to superficial zone injury, and various research results have shown that superficial injury is the key event that accelerates the process of full‐thickness cartilage degeneration.^35^, ^36^, ^37^

Moreover, 1‐669aa were used to treat OA rats. The results revealed, compared with the ACLT + Ep.‐Ad. group, matrix vertical fissures in the middle zone and branched fissures were observed. Safranin O stain depletion was more severe, and cell numbers were significantly decreased in the ACLT+1‐669aa group (Figure 2F), and the ACLT+1‐669aa group had higher OARSI scores (Figure 2G). These results indicated that 1‐669aa could cause the destruction of articular cartilage.

### 3.3 Differential gene expression and GO enrichment analysis

To determine the molecular mechanism by which the 1‐669aa (H4_N) affects chondrocytes and cartilage tissues, human chondrocytes were harvested and transfected with plasmids carrying EP, H4_N or full‐length HDAC4 (H4) for forty‐eight hours; then, the cells were subjected to RNA sequencing. The results of Pearson correlation between samples showed that the samples passed quality inspection for further analysis (the closer the *r* ^2^ was to 1, the better the correlation) (Figure S3a). Volcano plots were generated to compare the DEGs between H4_N and EP and those between H4 and EP, the results showed that both H4‐N (without the HDAC domain) and H4 (with the HDAC domain) induced significant changes in gene expression in chondrocytes (Figure S3b,c and Tables S1 and S2); these results further confirmed that 1‐669aa could exert biological effects independently of the HDAC domain.

**FIGURE 3:**
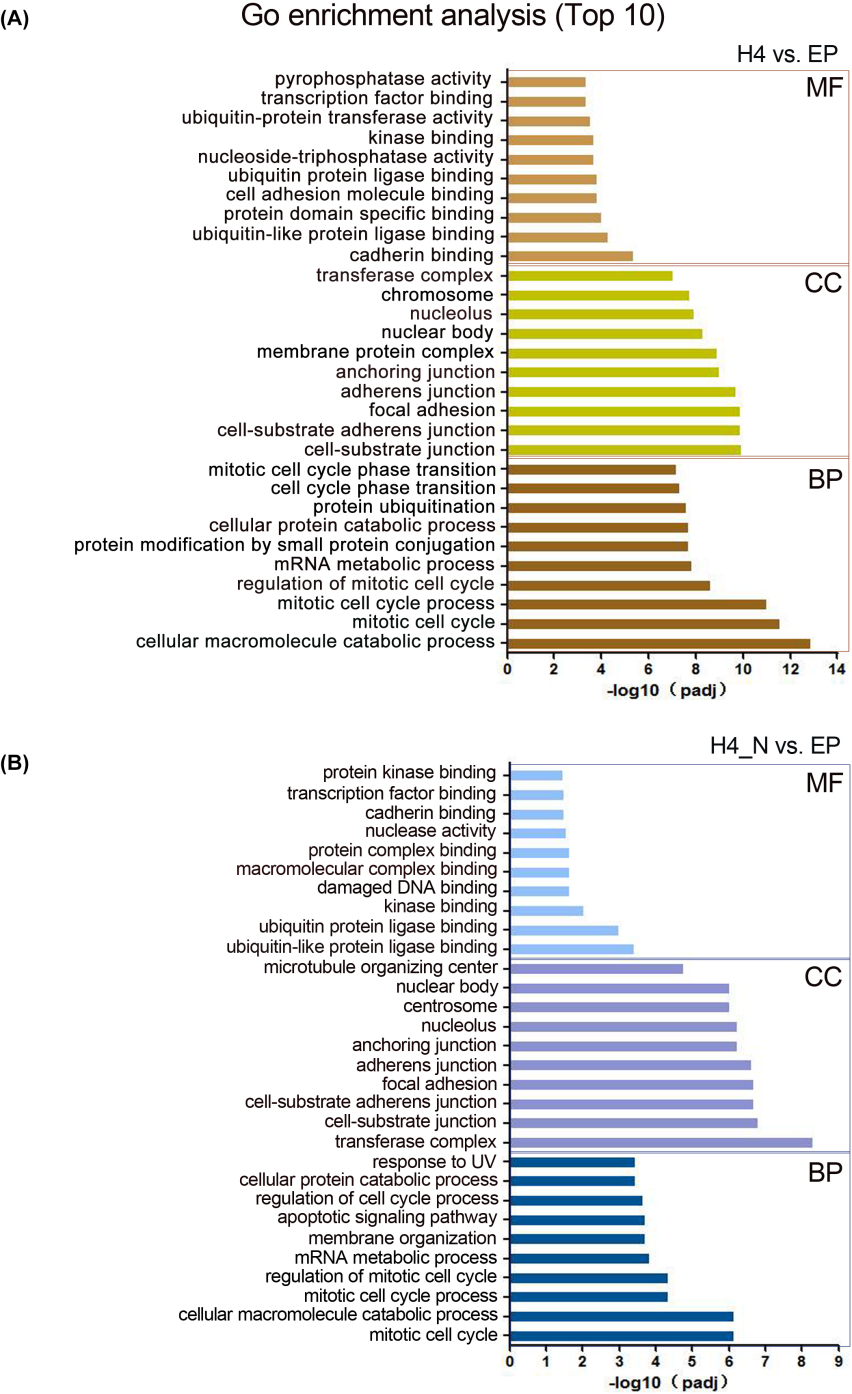
Gene Ontology (GO) functional enrichment analysis of DEGs for different treatment groups (H4 vs. EP and H4_N vs. EP). The terms with red lines below were mainly associated with the GO analysis of H4_N versus EP. BP, biological process; CC, cell component; EP, empty plasmid; H4, HDAC4; H4_N, HDAC4‐NT fragment 1‐669aa; MF, molecular function. A, the GO functional enrichment analysis of DEGs for H4 vs. EP; B, the GO functional enrichment analysis of DEGs for H4_N vs. EP.

Then, we performed GO enrichment analysis for the different treatment groups (H4 vs. EP and H4_N vs. EP). For both H4 versus EP and H4_N versus EP, GO analysis revealed multiple terms (*p* < 0.05; Tables S3 and S4). The top ten GO terms, including terms related to molecular function (MF), cellular component (CC) and biological process (BP), are shown in Figure 3A,B. Since the 1‐669aa fragment is a part of the H4 protein, we hypothesized that the biological functions of the fragment and the full‐length protein in chondrocytes may partially overlap but that the lack of an HDAC domain in the 1‐669aa fragment may cause its function to differ from that of the H4 protein. Therefore, we classified the two GO enrichment analyses described above, and the results showed that in the top 10 terms of BP, six terms overlapped both H4‐N versus EP and H4 versus EP, including mitotic cell cycle, cellular macromolecule catabolic process, mitotic cell cycle process, regulation of mitotic cell cycle, mRNA metabolic process and cellular protein catabolic process. Four terms were identified in the GO analysis of H4 vs. EP, mainly protein modification by small protein conjugation, cell cycle phase transition, mitotic cell cycle phase transition and protein ubiquitination. Importantly, four terms, namely, membrane organization, apoptotic signalling pathway, response to UV and regulation of cell cycle process, were mainly associated with the GO analysis of H4_N versus EP, and among these terms, apoptosis was an important cell death pathway (Figure S4a).

Additionally, a similar categorical analysis was carried out for the top 10 terms related to CC and MF. The results showed that in the CC analysis, H4_N was chiefly enriched in the terms centrosome and microtubule organizing center (Figure S4b), and in the MF analysis, H4_N was mostly enriched in various protein/DNA binding terms, such as damaged DNA binding, macromolecular complex binding, protein complex binding, and protein kinase binding (Figure S4c); these findings are consistent with the findings of other studies showing that H4 has a long N‐terminal region that is involved in interactions with different factors.^38^ Moreover, MF analysis revealed that 1‐669aa might regulate nuclear activity, which is associated with apoptosis.

At the same time, the apoptotic signalling pathway‐related gene for EP versus 1‐669aa were analysed. The volcano plot of the DEGs showed that 1‐669aa induced 185 significant gene expression changes (119 genes up‐regulated and 66 genes down‐regulated) (Figure S5a). Heat map showed the top 45 DEGs including 25 genes up‐regulated and 20 genes down‐regulated (Figure S5b). GSEA was performed too, and the results showed the apoptotic signalling pathway was up‐regulated (Figure S5c).

### 3.4 KEGG pathway analysis of DEGs between the 1‐669aa vs. EP groups and the H4 vs. EP groups

**FIGURE 4:**
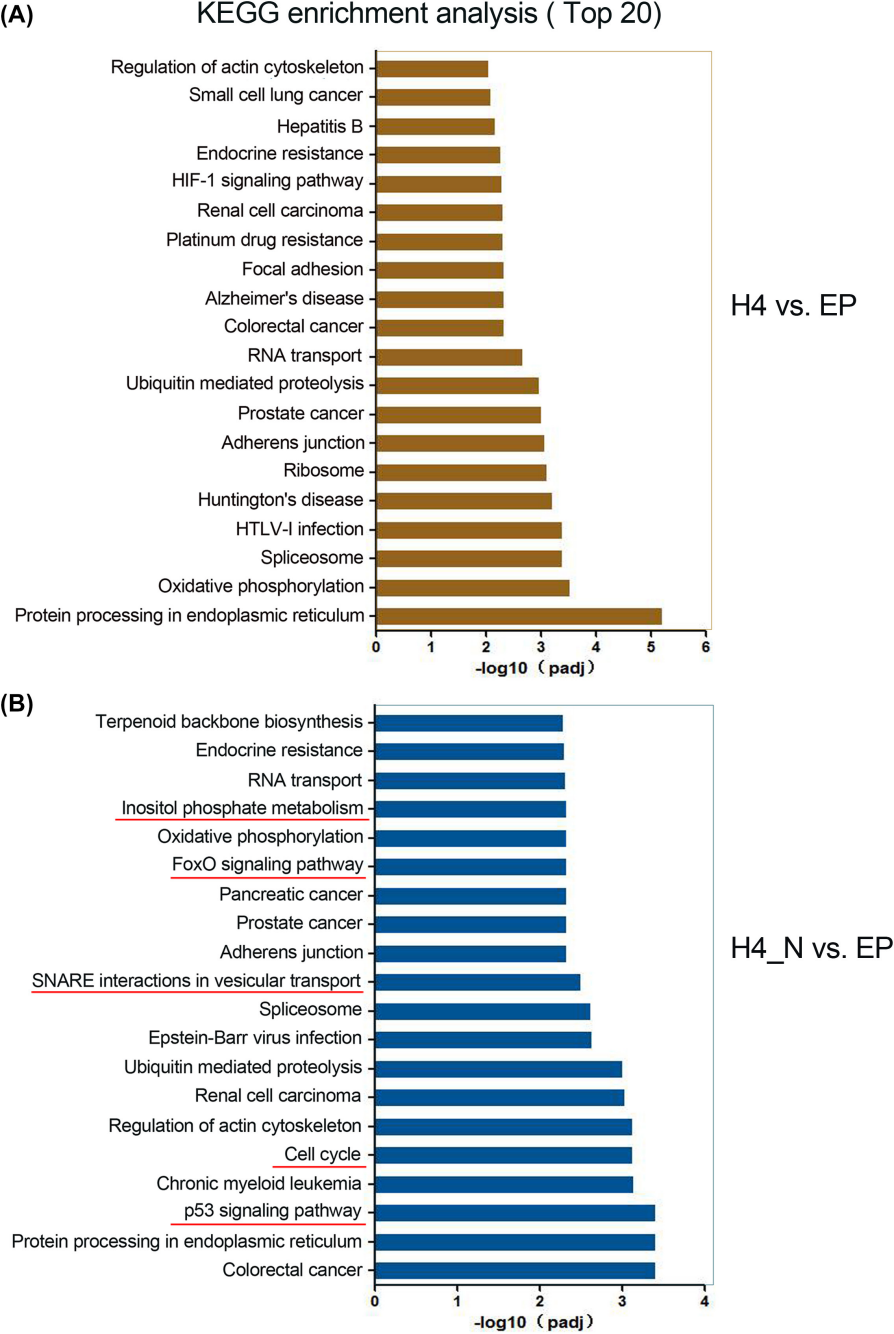
Kyoto Encyclopaedia of Genes and Genomes (KEGG) enrichment analysis of DEGs for different treatment groups (H4 vs. EP and H4_N vs. EP). The terms with red lines below were mainly associated with the KEGG analysis of H4_N versus EP. H4H4, HDAC4; EP, empty plasmid; H4_N, HDAC4‐NT fragment 1‐669aa. A, the GO functional enrichment analysis of DEGs for H4 vs. EP; B, the GO functional enrichment analysis of DEGs for H4_N vs. EP.

The Kyoto Encyclopaedia of Genes and Genomes (KEGG) database showed that for H4 versus EP, 56 pathways were identified (*p* < 0.05; Table S5), and for H4_N versus EP, 63 pathways were identified (*p* < 0.05; Table S6). The top twenty KEGG pathways are shown in Figure 4A,B, and a similar categorical analysis to that of GO enrichment analysis was performed. The results showed that eight signalling pathway terms were affected in both the H4_N and H4 groups, three pathways were primarily affected in the H4 group, and five pathways were primarily affected in the H4_N group, including the p53 signalling pathway, cell cycle, SNARE interactions in vesicular transport, FoxO signalling pathway, and inositol phosphate metabolism (Figure 4 and Figure S6). Among the five pathways, the p53 signalling pathway, which is an important pathway that promotes apoptosis, exhibited the most significant change. Thus, we inferred that the 1‐669aa‐induced apoptosis of chondrocytes may be closely related to the p53 signalling pathway.

### 3.5 In vitro validation of the RNA‐seq results

**FIGURE 5:**
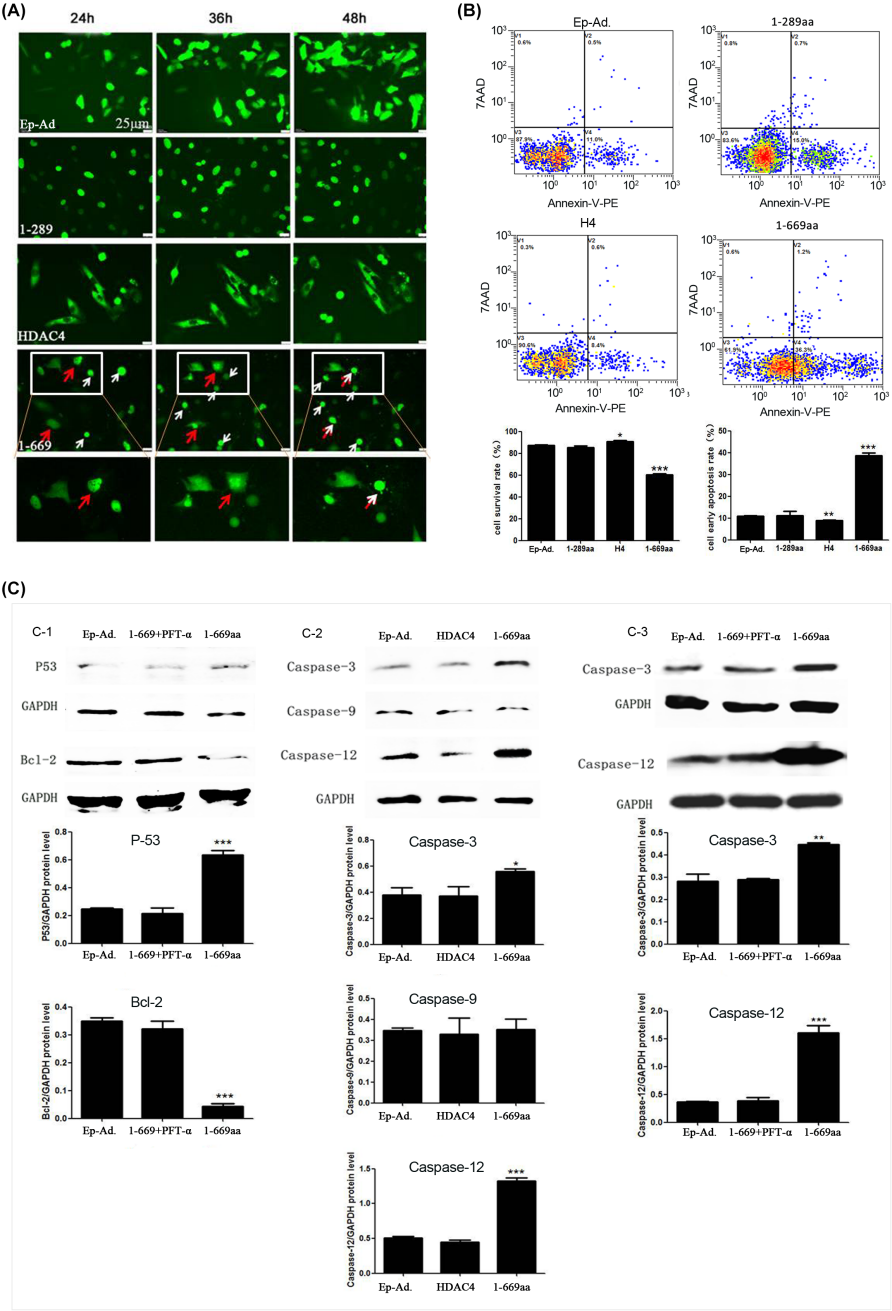
In vitro validation of the RNA‐seq. results. (A) Representative confocal microscopy images of different transfection groups (Ep.‐Ad., 1‐289aa, H4 and 1‐669aa) from 24 to 48 h after transfection, (Scale bar = 25 μm), white arrows showed the floating dead cells, red arrows showed the obvious nuclear cleavage. (B) Cell viability was detected by flow cytometry, and the data were quantified by the survival rate and the early apoptosis rate (*n* = 6). (C) Validation of p53, Bcl‐2, caspase‐3, caspase‐9 and caspase 12 expression by Western blotting (*n* = 3). Data are expressed as the mean ± SD, *p < 0.05; **p < 0.01, ***p < 0.001.

To validate the previous in vitro results and RNA‐seq results, Ep‐Ad. and 1‐289aa, 1‐669aa and H4 adenoviral DNA vectors were constructed, and ATDC5 chondrocytes were separately treated with these vectors. The cells were subjected to real‐time live‐cell imaging monitoring via a live cell real‐time monitoring system (TCS‐SP8; Leica, Germany) for 24 h (from 24 to 48 h after transfection) at 15‐min time intervals. Images were captured at 24, 36 and 48 h. As shown in the images, no significant cell death was observed in the Ep‐Ad., 1‐289aa and H4 transfection groups; however, in the 1‐669aa group, floating dead cells (white arrows) and obvious nuclear cleavage (red arrows) were observed (Figure 5A). Additionally, flow cytometry analysis of cell viability and apoptosis in the different groups after 48 h showed that 1‐669aa significantly promoted cell apoptosis and decreased cell viability (Figure 5B). Western blotting analysis also revealed that caspase‐3 expression was increased in the 1‐669aa group compared with the Ep‐Ad and H4 groups (Figure 5C‐2). These results confirmed that 1‐669aa could induce chondrocyte apoptosis.

To verify whether the induction of chondrocyte apoptosis by 1‐669aa was associated with the p53 signalling pathway, cells were randomly divided into the Ep‐Ad. and 1‐669aa groups. The protein expression of p53 and Bcl‐2 (a key anti‐apoptosis protein) was measured. The results showed that 1‐669aa could significantly increase the protein expression of p53 and decrease the expression of Bcl‐2 (Figure 5C‐1).

The intrinsic pathway, extrinsic pathway and endoplasmic reticulum stress (ERS) pathway are three important signalling pathways that induce apoptosis in cells, and caspase‐9, caspase‐8 and caspase‐12 are the key proteins of the intrinsic, extrinsic and ERS pathways, respectively.^39^ Studies have shown that p53 can induce apoptosis by activating caspase‐8 or caspase‐9.^40^, ^41^ Therefore, we measured the expression of these three caspase proteins and results showed that compared with Ep‐Ad. and H4, 1‐669aa could significantly increase the expression of caspase‐12, but there was no significant difference in caspase‐9. While caspase‐8 expression was very low, and no protein band was detected; therefore, the results are not shown in the figure (Figure 5C‐2). Inhibiting the expression of p53 via PFT‐α effectively decreased the expression of caspase‐12 and caspase‐3 (Figure 5C‐3). The results indicated that 1‐669aa might induce chondrocyte apoptosis via the ERS pathway, and upregulation of p53 is necessary for this process.

To validate the role of the ERS pathway in chondrocyte apoptosis induced by 1‐669aa, we analysed the ERS‐related genes for EP versus 1‐669aa, The volcano plot of the DEGs showed that 1‐669aa induced 99 significant gene expression changes (73 genes up‐regulated and 26 genes down‐regulated) (Figure S7a). Heat map showed the top 45 DEGs, including 32 genes up‐regulated and 13 genes down‐regulated (Figure S7b). GSEA results showed the term of the response to endoplasmic reticulum stress was up‐regulated significantly (Figure S7c). According to the volcano plot results of ERS‐related genes, the top1 DEGs HSPA5 (Figure S8a), also called GRP78, was validated by Western blotting. At the same time, according to the literature, the ERS‐related factor GRP94 was validated by Western blotting. The results showed that GRP94 had no change between EP‐Ad. and 1‐669aa groups, but GRP78 was increased in the HDAC4‐NT (1‐669aa) group, which is consistent with the RNA‐seq. analysis (Figure S8b).

### 3.6 In vivo validation of the RNA‐seq results

**FIGURE 6:**
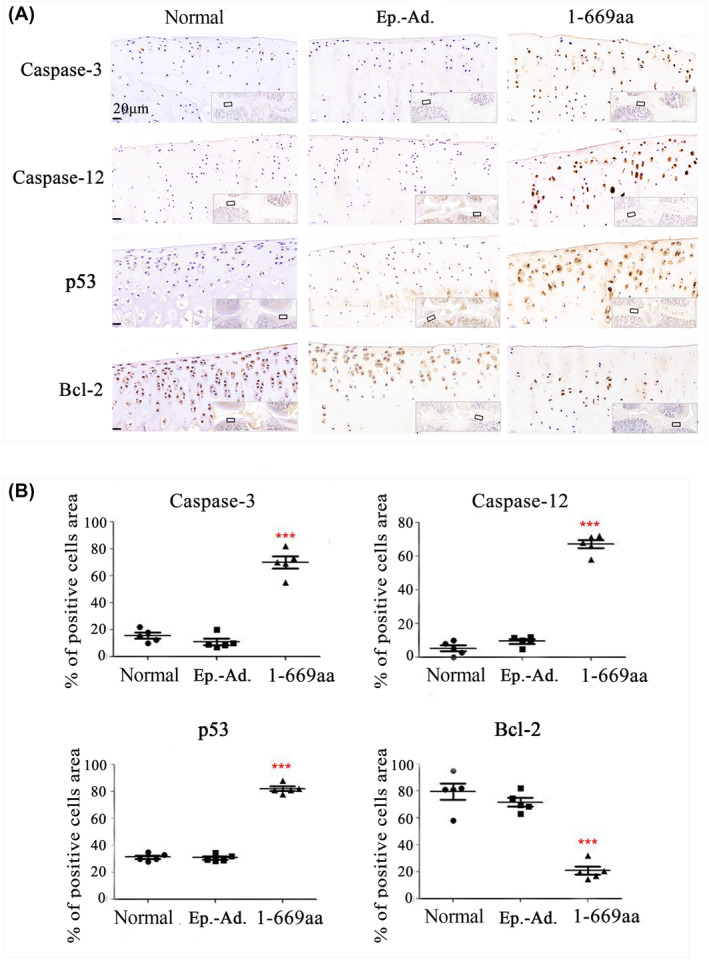
In vivo validation of the RNA‐seq. results. (A) The protein level of Caspase‐3, Caspase‐12, p53 and Bcl‐2 in the cartilage tissue of normal, normal + Ep.‐Ad. and normal +1‐669aa adenovirus DNA groups was detected by immunohistochemistry. (B) The data were quantified by the percentage (%) of positive cell area (*n* = 5), Data are expressed as the mean ± SD, ***p < 0.001.

**FIGURE 7:**
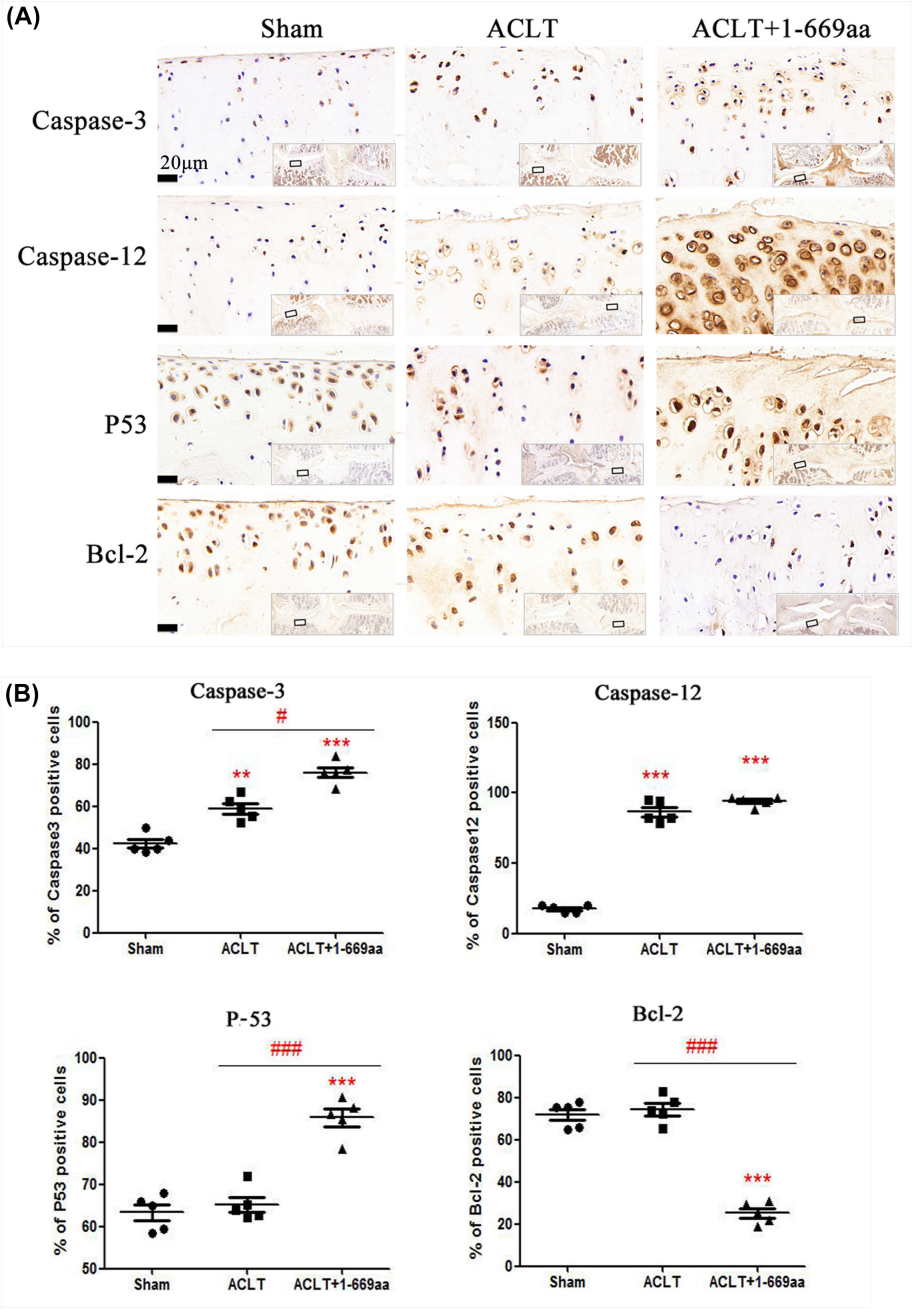
In vivo validation of the RNA‐seq. results. (A) The protein level of Caspase‐3, Caspase‐12, p53 and Bcl‐2 in the cartilage tissue of sham, ACLT + Ep.‐Ad. and ACLT +1‐669aa adenovirus DNA groups was detected by immunohistochemistry. (B) The data were quantified by the percentage (%) of positive cell area (*n* = 5), Data are expressed as the mean ± SD, **p < 0.01. ***p < 0.001 versus shame, ^#^ *p* < 0.05, ^###^ *p* < 0.001 versus ACLT.

To further validate the RNA‐seq results, we assayed the expression of caspase‐3, caspase‐12, p53 and Bcl‐2 in the cartilage tissues of normal rats and rats treated with 1‐289aa or 1‐669aa by immunohistochemistry. The results showed that compared to those in the normal and Ep.‐Ad. groups, the caspase‐3, caspase‐12 and p53 protein levels were increased whereas the Bcl‐2 protein levels were decreased in the 1‐669aa group (Figure 6A,B). Furthermore, the expression of the above proteins in the sham, ACLT and ACLT+1‐669aa groups was measured, and the results showed that 1‐669aa could significantly promote the protein expression of caspase‐3, caspase‐12 and p53 and reduce the protein expression of Bcl‐2 in cartilage tissue (Figure 7A,B). In vivo experiments showed the same result: 1‐669aa induced chondrocyte apoptosis by activating the p53‐dependent ERS pathway of apoptosis.

## 4 DISCUSSION

HDAC4 contains two distinct domains: the highly conserved C‐terminal domain is associated with HDAC catalytic activity, and the amino‐terminal domain (HDAC4‐NT) mediates protein–protein interactions with target factors as well as responses that modulate a variety of signalling pathways. Both domains play crucial roles in the function of HDAC4.^42^ Recently, studies have shown that the HDAC4‐NT that are produced by the degradation of HDAC4 also performs some biological functions^12^, ^13^, ^14^, ^15^, ^16^, ^17^, ^18^, ^43^; however, its function in chondrocytes and cartilage has not been determined. Here, we explored the function of HDAC4‐NT (1‐289aa, 1‐326aa and 1‐669aa) in chondrocytes and cartilage. The results showed that the HDAC4‐NT fragment 1‐669aa promoted chondrocyte death and cartilage degeneration via the p53‐dependent ERS pathway. This finding suggested that in addition to overexpressing HDAC4, preventing HDAC4 degradation may be a new strategy for the treatment of OA.

### 4.1 Selection of HDAC4‐NT fragments in this study

The crystal structure analysis of HDAC4‐NT showed that the domain included a glutamine‐rich motif (residues 62–153) that is believed to mediate the protein–protein interactions of HDAC4.^44^ A long list of HDAC4 partners has been identified; for example, transcription factors in the MEF2 family of bind to amino acids 118–180 of HDAC4,^21^ the Runx2‐binding domain of HDAC4 is located in the first 220 aa of the protein,^4^ the E1A C‐terminal binding protein (CtBP) interacts with HDAC4 via the P‐X‐D‐L‐R motif,^45^ calmodulin binding transcription activator binds to amino acids 150–220 of HDAC4,^23^ and more. These factors are closely associated with the biological and pathological processes of chondrocytes and cartilage, and the binding sites of these proteins in the HDAC4 protein are concentrated between amino acids 1 and 289. In IMR90‐E1A and IMR90‐E1A/C9DN cells, the 1‐289aa fragment is able to trigger cell death in a caspase‐9‐dependent manner, acting as a strong repressor of the transcription factor MEF2C.^17^ This fragment also can lead to markedly increased apoptosis via the repression of the serum response factors and Runx2 in IMR90‐E1A and U2OS cells.^16^ However, Paroni G discovered that the 1‐289aa fragment exhibited reduced repression of MEF2C‐driven transcription; thus, the researchers believed that PTMs might be one of the reasons for this discrepancy.^16^

PTMs regulate HDAC4 by controlling its subcellular localization, and the PTMs include the phosphorylation and dephosphorylation at S246, S467, S632, S265 and S266^22^, ^46^; sumoylation at K559^47^; and disulfide bond formation at C667/C669.^48^ Because HDAC4‐NT 1‐669aa includes almost all the important binding sites and PTM sites of HDAC4, in this study, three fragments were set: 1‐289aa, 1‐326aa, and 1‐669aa. The results showed that unlike its role in IMR90‐E1A and U2OS cells, the 1‐289aa could not promote chondrocyte death. However, 1‐669aa exhibited remarkable cell death‐promoting activity (Figures 1 and 2).

Furthermore, despite the Asp289 site is considered an important caspase‐dependent cleavage site in the HDAC4 protein,^12^ we previously detected HDAC4 fragments in normal and OA human cartilage tissues. The results showed that there were at least two kinds of HDAC4‐NT fragments in cartilage; one was located between 40 and 50 kD, which was close to the length of the 1‐289aa, and another was located between 70 and 100 kD, which was close to the length of the 1‐669aa fragment (Figure S9). Moreover, we also observed an approximately 90‐kDa band corresponding to degraded HDAC4‐NT in other related studies.^12^, ^16^, ^17^ These results suggested that except for the 289aa site, there may be other cleavage sites in HDAC4 that generate the longer HDAC4‐NT fragment, and the longer fragment of HDAC4‐NT may perform biological functions because it retains various binding sites and PTM sites.

### 4.2 Exploring the molecular mechanism of 1‐669aa induced chondrocyte death

To investigate the molecular mechanism by which 1‐669aa decreases the survival of chondrocytes, RNA‐seq was performed to compare 1‐669aa vs. EP and HDAC4 vs. EP. Our results showed that according to the GO analysis, the DEGs whose expression was altered by 1‐669aa were enriched mainly in the apoptotic signalling pathway. According to the KEGG analysis, the DEGs were enriched mainly in the p53 signalling pathway. These results suggested that p53 may be involved in the induction of chondrocyte apoptosis by 1‐669aa (Figures 3 and 4). Through in vitro and in vivo experiments (Figures 5, 6, 7), we further confirmed that 1‐669aa induced chondrocyte apoptosis, increasing the protein expression of p53.

P53 is a key proapoptotic protein, and studies have shown that p53 can not only activate caspase‐8 and induce apoptosis but also activate caspase‐9 and induce apoptosis via the cytochrome C pathway.^40^, ^41^ Therefore, this study examined the expression of caspase‐8 and caspase‐9. However, the results showed that the expression of caspase‐9 in the 1‐669aa group did not be significantly changed; the expression of caspase‐8 was very low, and no protein band was detected. Then, we examined the expression of caspase‐12, which is a key protein that is involved in ERS‐induced apoptosis (Figure 5C). The results showed that 1‐669aa significantly increased the expression of caspase‐12 in chondrocytes. ERS may be an important pathway that is involved in 1‐669aa‐induced chondrocyte apoptosis.

The relationship between p53 and ERS is complex, and several studies have shown that ERS can inhibit p53.^49^, ^50^ Jianze Li et al. showed that p53 is a novel component of the ERS‐induced apoptotic pathway and contributes to ERS‐induced apoptosis.^51^ Limeng Dai et al. suggested that TSA‐induced cell apoptosis is dependent on p53.^52^ Wan‐Chi Lin et al. also showed that NF‐kB activation and p53 expression induction are essential for ERS‐induced cell death.^53^ To investigate the role of p53 in the ERS pathway‐dependent chondrocyte apoptosis induced 1‐669aa, this study used a p53 inhibitor (PFT‐α) to treat cells. The results showed that inhibiting p53 expression could significantly suppress the 1‐669aa‐induced expression of caspase‐12 and caspase‐3 (Figure 5C‐3), which revealed that 1‐669aa may induce apoptosis via a p53‐dependent ERS pathway in chondrocytes.

In conclusion, different from the role of inducing osteosarcoma cell apoptosis, HDAC4‐NT of 1‐289aa has no obvious effect in chondrocytes and cartilage tissue, but HDAC4‐NT of 1‐669aa can induce chondrocyte apoptosis via the p53‐dependent ERS pathway, which may be related to the inclusion of almost all the important binding sites and PTM sites of HDAC4 in the 1‐669aa segment. However, it remains unclear where is the functional site of 1‐669aa inducing chondrocyte apoptosis.

## AUTHOR CONTRIBUTIONS

**Li Guo:** Conceptualization (lead); data curation (lead); formal analysis (lead); funding acquisition (lead); writing – original draft (lead); writing – review and editing (lead). **Xuhao Zhuo:** Validation (equal); writing – review and editing (equal). **Chengyang Lu:** Formal analysis (equal); writing – review and editing (equal). **Hua Guo:** Data curation (equal); methodology (equal); validation (equal). **Zhi Chen:** Funding acquisition (lead); resources (equal). **Gaige Wu:** Data curation (equal); methodology (equal). **Fengrui Liu:** Methodology (equal). **Xiaochun Wei:** Funding acquisition (lead); project administration (equal); supervision (equal). **Xueqin Rong:** Funding acquisition (equal). **Pengcui Li:** Conceptualization (equal); funding acquisition (lead); project administration (lead); writing – review and editing (equal).

## FUNDING INFORMATION

This work was supported by grants from the Regional Innovation Joint Fund of the National Natural Science Foundation of China (Integrated Project) (U23A6009), the Regional Innovation Joint Fund of the National Natural Science Foundation of China (Key Project) (U21A20353), National Natural Science Foundation of China (82172503), the Natural Science Foundation of Shanxi Province (20210302123285, 20210302123263 and 20210302123283), the Key R&D Program Projects of Shanxi Province (202202040201012) and the Hainan Provincial Medical and Health Research Program (21A200349).

## CONFLICT OF INTEREST STATEMENT

The authors of this manuscript declare no relationships with any companies whose products or services may be related to the subject matter of the article.

## Supporting information


Figure S1.

Table S1.

## Data Availability

All the data in this study are presented in the manuscript and supplementary materials.

## References

[1] Minnig MCC , Golightly YM , Nelson AE . Epidemiology of osteoarthritis: literature update 2022‐2023. Curr Opin Rheumatol. 2024;36(2):108‐112. 38240280 10.1097/BOR.0000000000000985 PMC10965245

[2] Peat G , Thomas MJ . Osteoarthritis year in review 2020: epidemiology & therapy. Osteoarthr Cartil. 2021;29(2):180‐189. 10.1016/j.joca.2020.10.007 33242603

[3] Hou F , Wei W , Qin X , et al. The posttranslational modification of HDAC4 in cell biology: mechanisms and potential targets. J Cell Biochem. 2020;121(2):930‐937. 31588631 10.1002/jcb.29365

[4] Martin SD , Connor T , Sanigorski A , et al. Class IIa HDACs inhibit cell death pathways and protect muscle integrity in response to lipotoxicity. Cell Death Dis. 2023;14(12):787. 38040704 10.1038/s41419-023-06319-5 PMC10692215

[5] Vega RB , Matsuda K , Oh J , et al. Histone deacetylase 4 controls chondrocyte hypertrophy during skeletogenesis. Cell. 2004;119(4):555‐566. 15537544 10.1016/j.cell.2004.10.024

[6] Dong Z , Ma Z , Yang M , et al. The level of histone deacetylase 4 is associated with aging cartilage degeneration and chondrocyte hypertrophy. J Inflamm Res. 2022;15:3547‐3560. 35734099 10.2147/JIR.S365545 PMC9208673

[7] Chen Z , Zhang Z , Guo L , et al. The role of histone deacetylase 4 during chondrocyte hypertrophy and endochondral bone development. Bone Joint Res. 2020;9(2):82‐89. 32435460 10.1302/2046-3758.92.BJR-2019-0172.R1 PMC7229302

[8] Cao K , Wei L , Zhang Z , et al. Decreased histone deacetylase 4 is associated with human osteoarthritis cartilage degeneration by releasing histone deacetylase 4 inhibition of runt‐related transcription factor‐2 and increasing osteoarthritis‐related genes: a novel mechanism of human osteoarthritis cartilage degeneration. Arthritis Res Ther. 2014;16(6):491. 25424126 10.1186/s13075-014-0491-3 PMC4265470

[9] Jensen ED , Nair AK , Westendorf JJ . Histone deacetylase co‐repressor complex control of runx2 and bone formation. Crit Rev Eukaryot Gene Expr. 2007;17(3):187‐196. 17725488 10.1615/critreveukargeneexpr.v17.i3.20

[10] Xu D , Gao Y , Hu N , Wu L . Chen Q.miR‐365 ameliorates dexamethasone‐induced suppression of Osteogenesis in MC3T3‐E1 cells by targeting HDAC4. Int J Mol Sci. 2017;18(5):977. 28471397 10.3390/ijms18050977 PMC5454890

[11] Gu XD , Wei L , Li PC , et al. Adenovirus‐mediated transduction with histone deacetylase 4 ameliorates disease progression in an osteoarthritis rat model. Int Immunopharmacol. 2019;75:105752. 31310910 10.1016/j.intimp.2019.105752

[12] Liu F , Dowling M , Yang XJ , Kao GD . Caspase‐mediated specific cleavage of human histone deacetylase 4. J Biol Chem. 2004;279(33):34537‐34546. 15205465 10.1074/jbc.M402475200

[13] Backs J , Worst BC , Lehmann LH , et al. Selective repression of MEF2 activity by PKA‐dependent proteolysis of HDAC4. J Cell Biol. 2011;195(3):403‐415. 22042619 10.1083/jcb.201105063 PMC3206346

[14] Lehmann LH , Jebessa ZH , Kreusser MM , et al. A proteolytic fragment of histone deacetylase 4 protects the heart from failure by regulating the hexosamine biosynthetic pathway. Nat Med. 2018;24(1):62‐72. 29227474 10.1038/nm.4452

[15] Lv X , Tian X , Dong B , Wang H , Su X , Liu B . ABHD5 protects cardiac function in alcoholic cardiomyopathy via HDAC4‐NT to inhibit CAMKII/MEF2 axis. J Appl Physiol (1985). 2024;136(5):1291. doi: 10.1152/japplphysiol.00329.2023_RET 38743613

[16] Paroni G , Fontanini A , Cernotta N , et al. Dephosphorylation and caspase processing generate distinct nuclear pools of histone deacetylase 4. Mol Cell Biol. 2007;19:6718‐6732. 10.1128/MCB.00853-07 PMC2099224 17636017

[17] Paroni G , Mizzau M , Henderson C , Del Sal G , Schneider C , Brancolini C . Caspase‐dependent regulation of histone deacetylase 4 nuclear–cytoplasmic shuttling promotes apoptosis. Mol Biol Cell. 2004;15(6):2804‐2818. 15075374 10.1091/mbc.E03-08-0624 PMC420104

[18] Rajan I , Savelieva KV , Ye GL , et al. Loss of the putative catalytic domain of HDAC4 leads to reduced thermal nociception and seizures while allowing normal bone development. PLoS One. 2009;4(8):e6612. 19672313 10.1371/journal.pone.0006612 PMC2720538

[19] Miska EA , Karlsson C , Langley E , Nielsen SJ , Pines J , Kouzarides T . HDAC4 deacetylase associates with and represses the MEF2 transcription factor. EMBO J. 1999;18:5099‐5107. 10487761 10.1093/emboj/18.18.5099 PMC1171580

[20] Chan JK , Sun L , Yang XJ , Zhu G , Wu Z . Functional characterization of an amino‐terminal region of HDAC4 that possesses MEF2 binding and transcriptional repressive activity. J Biol Chem. 2003;278:23515‐23521. 12709441 10.1074/jbc.M301922200

[21] Wang AH , Bertos NR , Vezmar M , et al. Yang XJ.HDAC4, a human histone deacetylase related to yeast HDA1, is atranscriptional corepressor. Mol Cell Biol. 1999;19(11):7816‐7827. 10523670 10.1128/mcb.19.11.7816 PMC84849

[22] Grozinger CM , Schreiber SL . Regulation of histone deacetylase 4 and 5 and transcriptional activity by 14‐3‐3‐dependent cellular localization. Proc Natl Acad Sci USA. 2000;97:7835‐7840. 10869435 10.1073/pnas.140199597 PMC16631

[23] Youn H‐D , Grozinger CM , Liu JO . Calcium regulatestranscriptional repression of myocyte enhancer factor 2 byhistone deacetylase 4. J Biol Chem. 2000;275:22563‐22567. 10825153 10.1074/jbc.C000304200

[24] Davis FJ , Gupta M , Camoretti‐Mercado B , Schwartz RJ , Gupta MP . Calcium/Calmodulin‐dependent protein kinase activates SerumResponse factor transcription activity by its dissociation fromHistone deacetylase, HDAC4. J Biol Chem. 2003;278(22):20047‐20058. 12663674 10.1074/jbc.M209998200

[25] Wang AH , Yang XJ . Histone deacetylase 4 possesses intrinsic nuclear import and export signals. Mol Cell Biol. 2001;21:5992‐6005. 11486037 10.1128/MCB.21.17.5992-6005.2001 PMC87317

[26] Backs J , Song K , Bezprozvannaya S , Chang S , Olson EN . CaM kinase II selectively signals to histone deacetylase 4 during cardiomyocyte hypertrophy. J Clin Invest. 2006;116:1853‐1864. 16767219 10.1172/JCI27438 PMC1474817

[27] Wei L , Sun X , Kanbe K , et al. Chondrocyte death induced by pathological concentration of chemokine stromal cell‐derived factor‐1. J Rheumatol. 2006;33:1818‐1826. 16960943

[28] Wei L , Sun XJ , Wang Z , Chen Q . CD95‐induced osteoarthritic chondrocyte apoptosis and necrosis: dependency on p38 mitogen‐activated protein kinase. Arthritis Res Ther. 2006;8:R37. 16469115 10.1186/ar1891 PMC1526592

[29] Luo Y , Gao X , Zou L , Lei M , Feng J , Hu Z . Bavachin induces Ferroptosis through the STAT3/P53/SLC7A11 Axis in osteosarcoma cells. Oxidative Med Cell Longev. 2021;2021:1783485. 10.1155/2021/1783485 PMC8545544 34707773

[30] Bunpetch V , Zhang X , Li T , et al. Silicate‐based bioceramic scaffolds for dual‐lineage regeneration of osteochondral defect. Biomaterials. 2019;192:323‐333. 30468999 10.1016/j.biomaterials.2018.11.025

[31] Jay GD , Fleming BC , Watkins BA , et al. Prevention of cartilage degeneration and restoration of chondroprotection by lubricin tribosupplementation in the rat following anterior cruciate ligament transection. Arthritis Rheum. 2010;62:2382‐2391. 20506144 10.1002/art.27550 PMC2921027

[32] Huang L , Li P , Guo L , et al. Zinc finger protein 521 attenuates osteoarthritis via the histone deacetylases 4 in the nucleus [J]. Bioengineered. 2022;13(6):14489‐14502. 36694467 10.1080/21655979.2022.2090203 PMC9995124

[33] Laferriere CA , Pang DS . Review of intraperitoneal injection of sodium pentobarbital as a method of euthanasia in laboratory rodents. J Am Assoc Lab Anim Sci. 2020;59(3):254‐263. 10.30802/AALAS-JAALAS-19-000081 PMC7210732 32156325

[34] Guo L , Guo H , Zhang Y , et al. Upregulated ribosome pathway plays a key role in HDAC4, improving the survival rate and biofunction of chondrocytes. Bone Joint Res. 2023;12(7):433‐446. 37414410 10.1302/2046-3758.127.BJR-2022-0279.R2 PMC10325875

[35] Si Y , Tan Y , Gao L , et al. Mechanical properties of cracked articular cartilage under uniaxial creep and cyclic tensile loading. J Biomech. 2022;134:110988. 35151037 10.1016/j.jbiomech.2022.110988

[36] Gannon AR , Nagel T , Bell AP , Avery NC , Kelly DJ . The changing role of the superficial region in determining the dynamic compressive properties of articular cartilage during postnatal development. Osteoarthr Cartil. 2015;23(6):975‐984. 10.1016/j.joca.2015.02.003 25680651

[37] Mäkelä JT , Han SK , Herzog W , Korhonen RK . Very early osteoarthritis changes sensitively fluid flow properties of articular cartilage. J Biomech. 2015;48(12):3369‐3376. 26159056 10.1016/j.jbiomech.2015.06.010

[38] Cuttini E , Goi C , Pellarin E , Vida R , Brancolini C . HDAC4 in cancer: a multitasking platform to drive not only epigenetic modifications. Front Mol Biosci. 2023;10:1116660. 36762207 10.3389/fmolb.2023.1116660 PMC9902726

[39] Huang R , Hui Z , Wei S , et al. IRE1 signaling regulates chondrocyte apoptosis and death fate in the osteoarthritis. J Cell Physiol. 2022;237(1):118‐127. 34297411 10.1002/jcp.30537 PMC9291116

[40] Guan HM , Li WQ , Liu J , Zhou JY . Corrigendum to "LncRNA HIF1A‐AS2 modulated by HPV16 E6 regulates apoptosis of cervical cancer cells via P53/caspase9/caspase3 axis" [cellular Signalling 97 (2022) 110390]. Cell Signal. 2023;102:110519. 36462269 10.1016/j.cellsig.2022.110519

[41] Nazmy EA , El‐Khouly OA , Zaki MMA , et al. Targeting p53/TRAIL/caspase‐8 signaling by adiponectin reverses thioacetamide‐induced hepatocellular carcinoma in rats. Environ Toxicol Pharmacol. 2019;72:103240. 31421311 10.1016/j.etap.2019.103240

[42] Fischle W , Kiermer V , Dequiedt F , Verdin E . The emerging role of class II histone deacetylases. Biochem Cell Biol. 2001;79(3):337‐348. 11467747

[43] Guo X , Wang SB , Xu H , et al. A short N‐terminal domain of HDAC4 preserves photoreceptors and restores visual function in retinitis pigmentosa. Nat Commun. 2015;6:8005. 26272629 10.1038/ncomms9005 PMC4538705

[44] Guo L , Han A , Bates DL , Cao J , Chen L . Crystal structureof a conserved N‐terminal domain of histone deacetylase 4 revealsfunctional insights into glutamine‐rich domains. Proc Natl Acad Sci USA. 2007;104(11):4297‐4302. 17360518 10.1073/pnas.0608041104 PMC1838596

[45] Zhang CL , McKinsey TA , Lu JR , Olson EN . Association of COOH‐terminal‐binding protein (CtBP) andMEF2‐interacting transcription repressor (MITR) contributes toTranscriptional repression of the MEF2 transcription factor. J Biol Chem. 2001;276(1):35‐39. 11022042 10.1074/jbc.M007364200

[46] Guise AJ , Greco TM , Zhang IY , Yu F , Cristea IM . Aurora B‐dependent regulation of class IIaHistone deacetylases by mitotic NuclearLocalization signal phosphorylation. Mol Cell Proteomics. 2012;11(11):1220‐1229. 22865920 10.1074/mcp.M112.021030 PMC3494195

[47] Kirsh O , Seeler JS , Pichler A , et al. The SUMO E3 ligase RanBP2 promotes modification of the HDAC4 deacetylase. EMBO J. 2002;21(11):2682‐2691. 12032081 10.1093/emboj/21.11.2682 PMC125385

[48] Ago T , Liu T , Zhai P , et al. Aredox‐dependent pathway for regulating class II HDACs and cardiac hypertrophy. Cell. 2008;133(6):978‐993. 18555775 10.1016/j.cell.2008.04.041

[49] Stavridi ES , Halazonetis TD . p53 and stress in the ER. Genes Dev. 2004;18(3):241‐244. 14871924 10.1101/gad.1181704

[50] Yamasaki S , Yagishita N , Nishioka K , Nakajima T . The roles of synoviolin in crosstalk between endoplasmic reticulum stress‐induced apoptosis and p53 pathway. Cell Cycle. 2007;6(11):1319‐1323. 17582219 10.4161/cc.6.11.4277

[51] Li J , Lee B , Lee AS . Endoplasmic reticulum stress‐induced apoptosis: multiple pathways and activation of p53‐up‐regulated modulator of apoptosis (PUMA) and NOXA by p53. J Biol Chem. 2006;281(11):7260‐7270. 16407291 10.1074/jbc.M509868200

[52] Dai L , He G , Zhang K , Guan X , Wang Y , Zhang B . Trichostatin a induces p53‐dependent endoplasmic reticulum stress in human colon cancer cells. Oncol Lett. 2019;17(1):660‐667. 30655814 10.3892/ol.2018.9641 PMC6313176

[53] Lin WC , Chuang YC , Chang YS , et al. Endoplasmic reticulum stress stimulates p53 expression through NF‐κB activation. PLoS One. 2012;7(7):e39120. 22859938 10.1371/journal.pone.0039120 PMC3408479

