# Supplementary figures and images for: The N‐terminal fragment of histone deacetylase 4 (1‐669aa) promotes chondrocyte apoptosis via the p53‐dependent endoplasmic reticulum stress pathway
Source: J Cell Mol Med. 2024 Oct 20;28(20):e70135. doi: 10.1111/jcmm.70135 (PMC11491302; doi:10.1111/jcmm.70135)

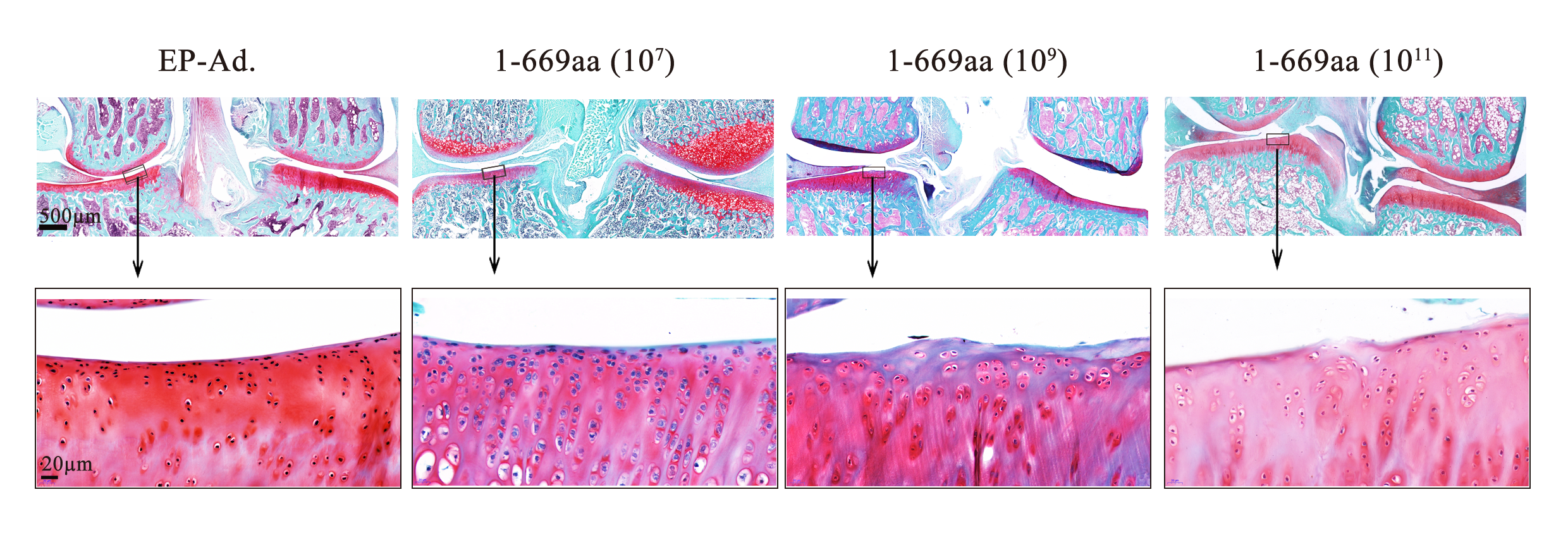

Supplement: Supplementary file 1 — Figure S1. [file JCMM-28-e70135-s002.zip › Figure-S-1.tif]

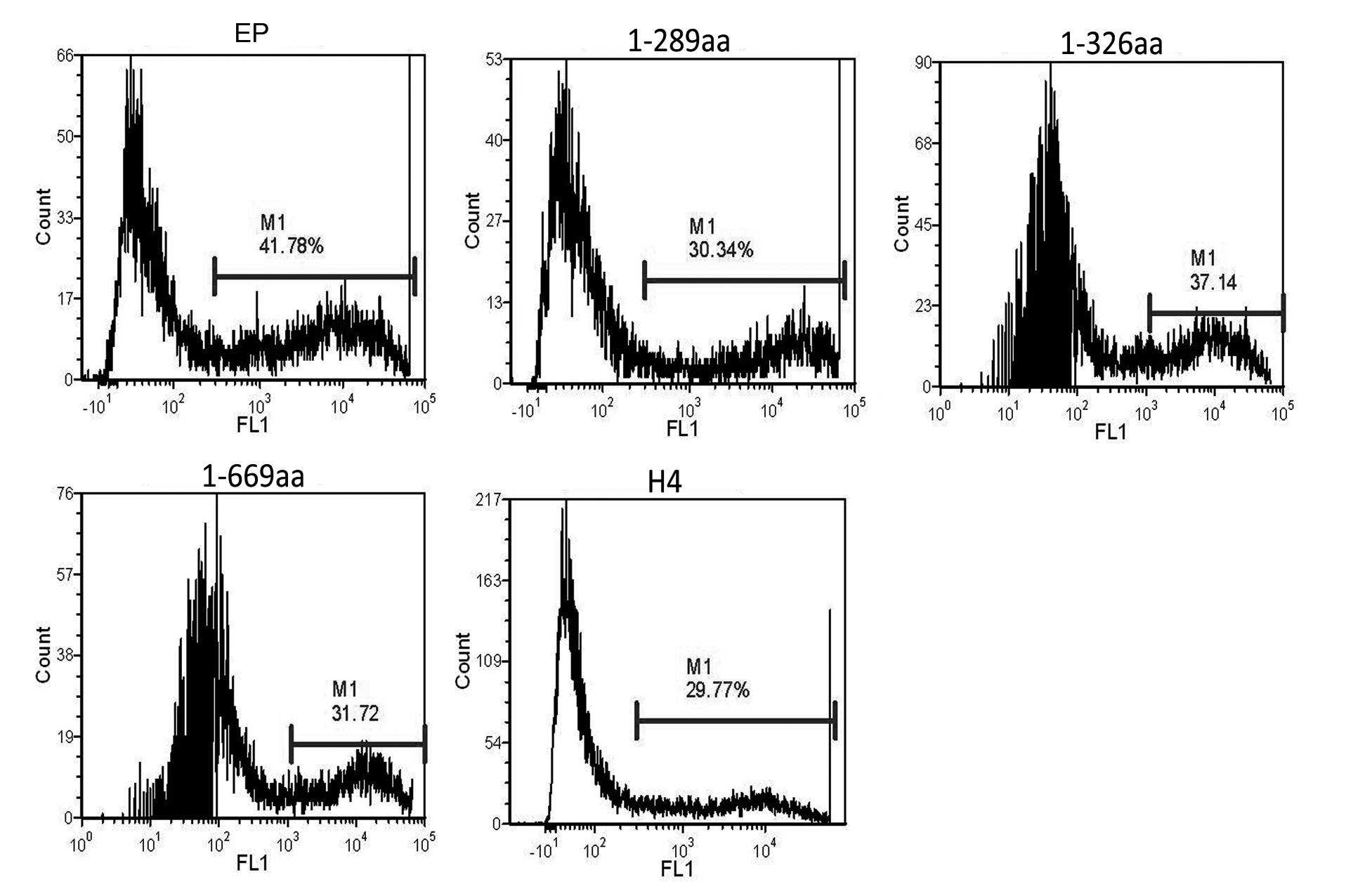

Supplement: Supplementary file 1 — Figure S1. [file JCMM-28-e70135-s002.zip › Figure-S-2.png]

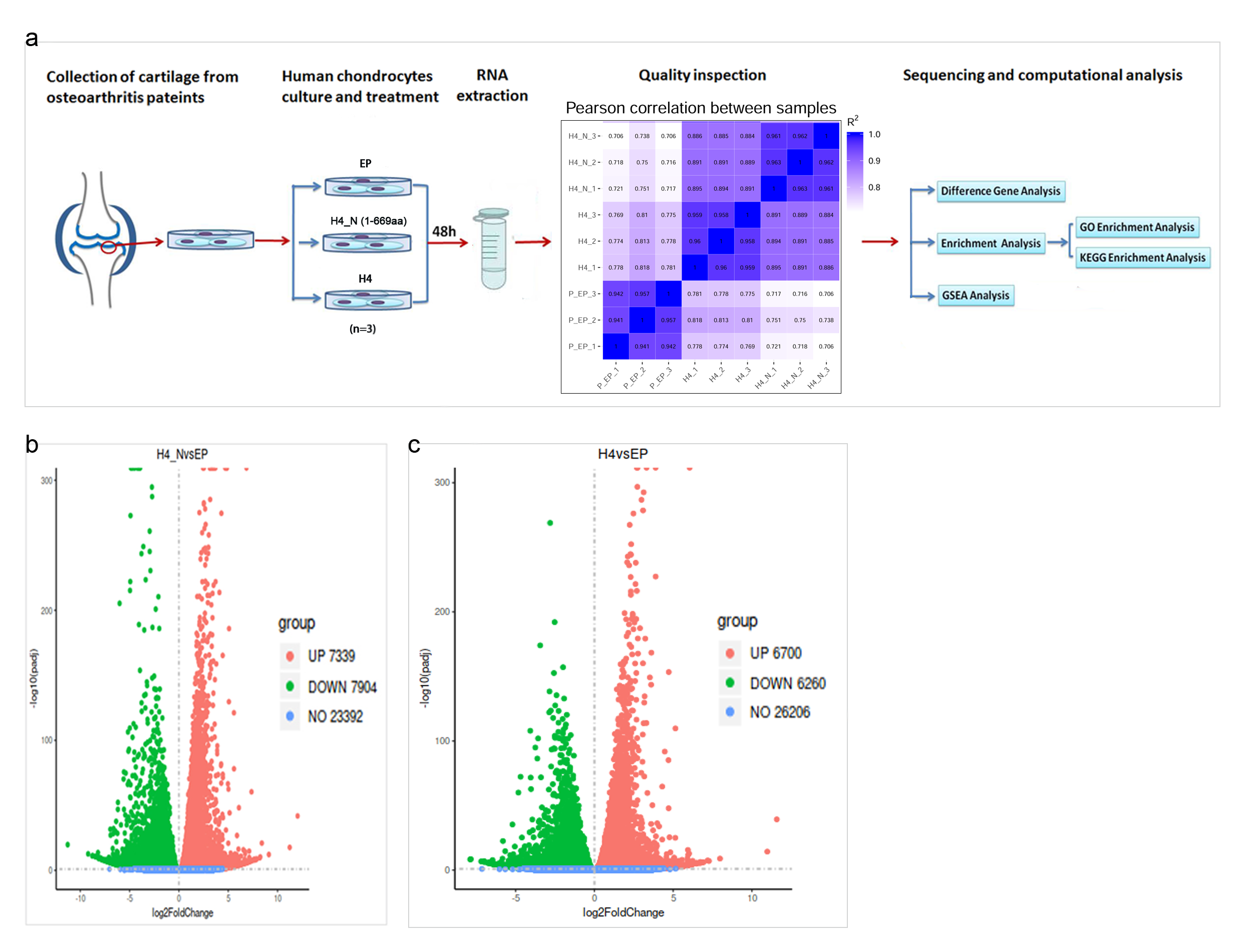

Supplement: Supplementary file 1 — Figure S1. [file JCMM-28-e70135-s002.zip › Figure-S-3 .png]

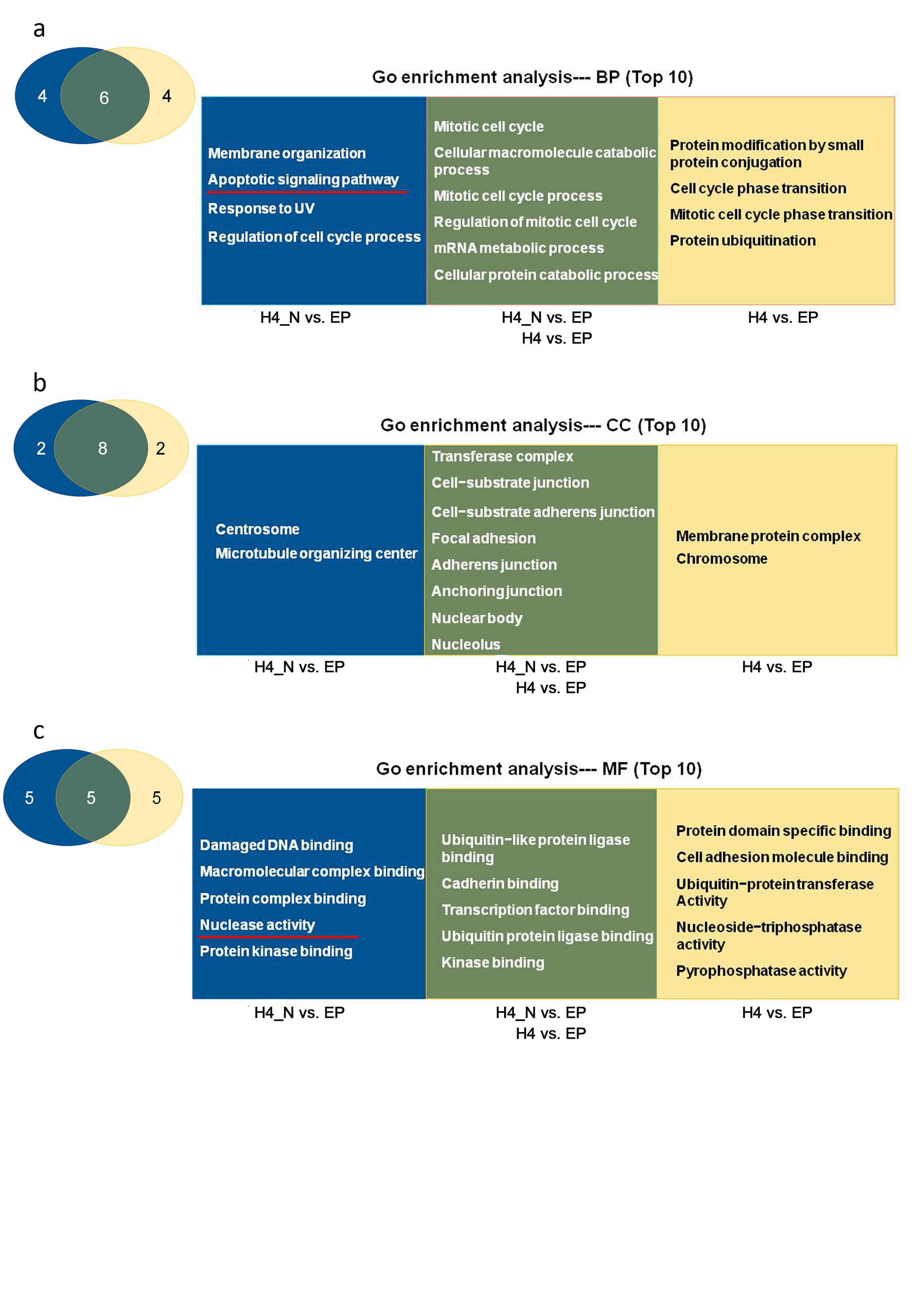

Supplement: Supplementary file 1 — Figure S1. [file JCMM-28-e70135-s002.zip › Figure-S-4.png]

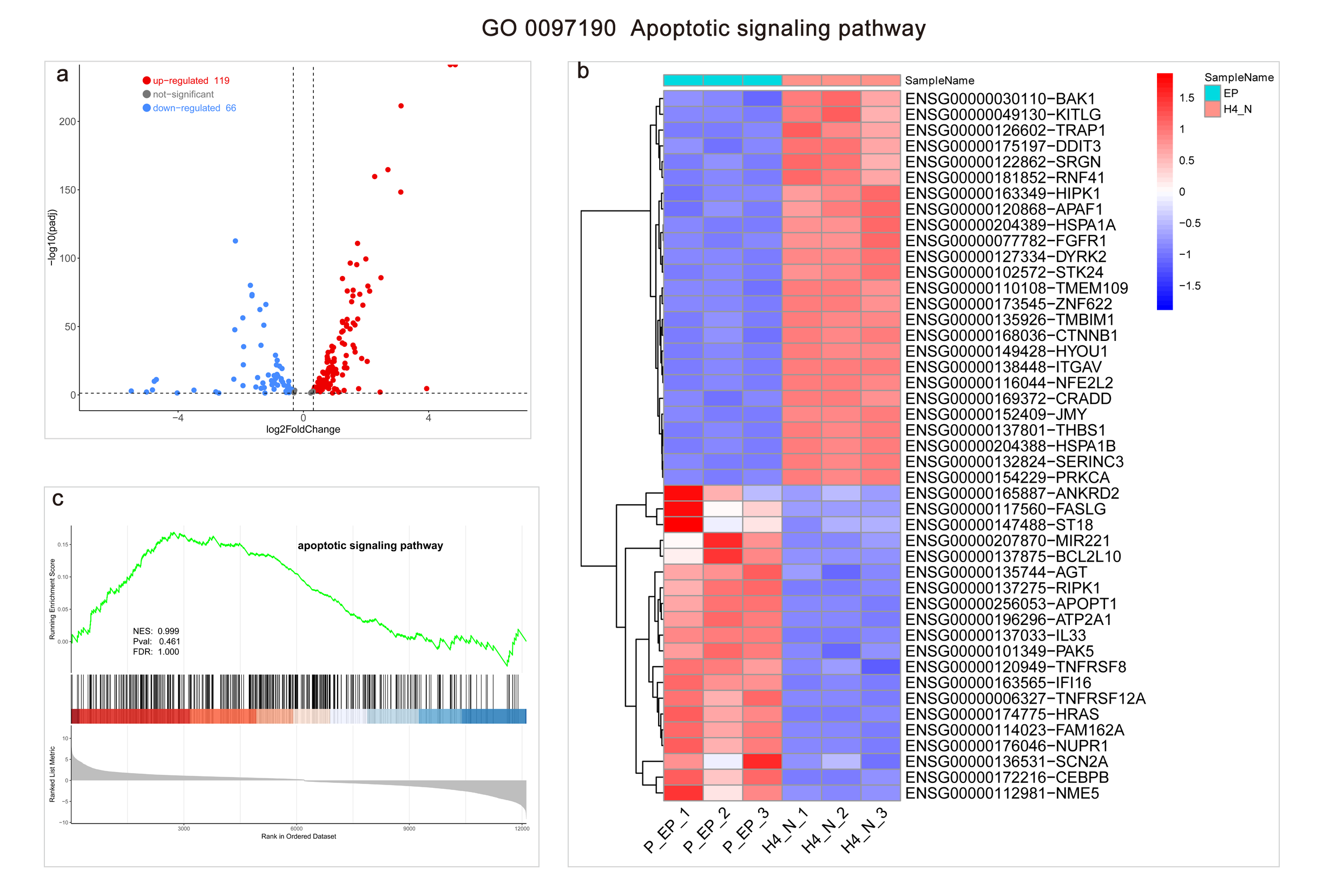

Supplement: Supplementary file 1 — Figure S1. [file JCMM-28-e70135-s002.zip › Figure-S-5 Apoptosis.png]

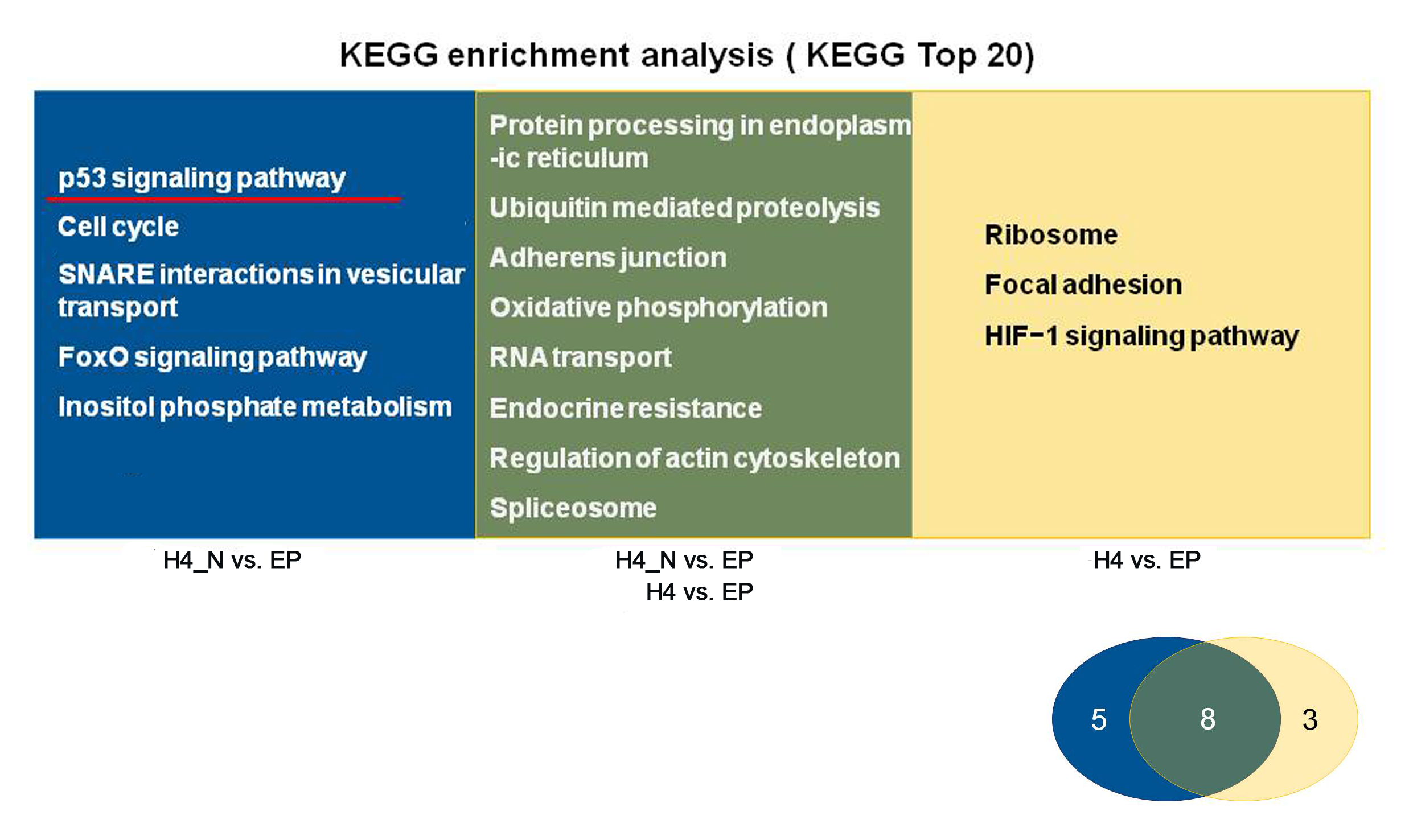

Supplement: Supplementary file 1 — Figure S1. [file JCMM-28-e70135-s002.zip › Figure-S-6.png]

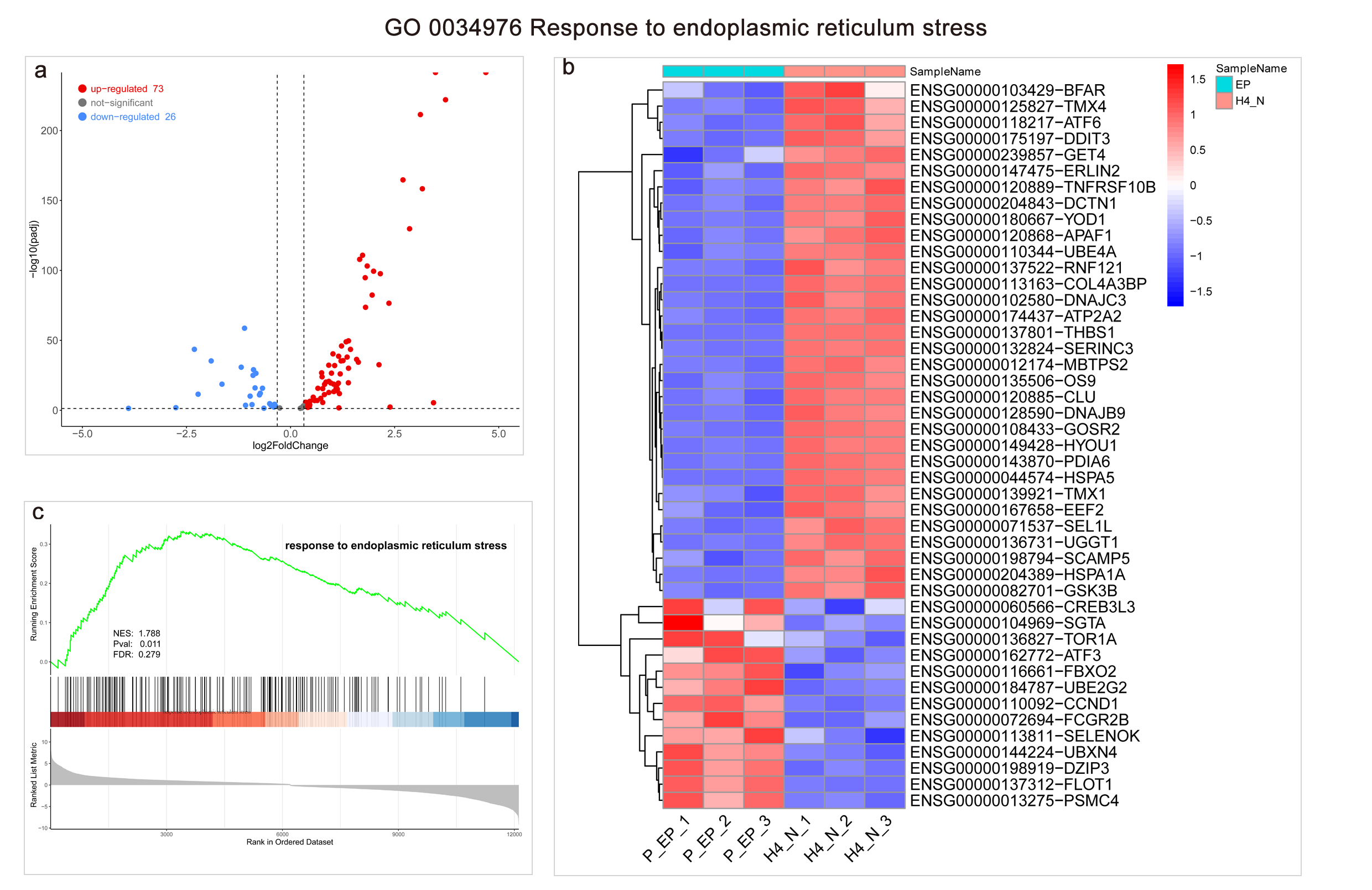

Supplement: Supplementary file 1 — Figure S1. [file JCMM-28-e70135-s002.zip › Figure-S-7 ERS.png]

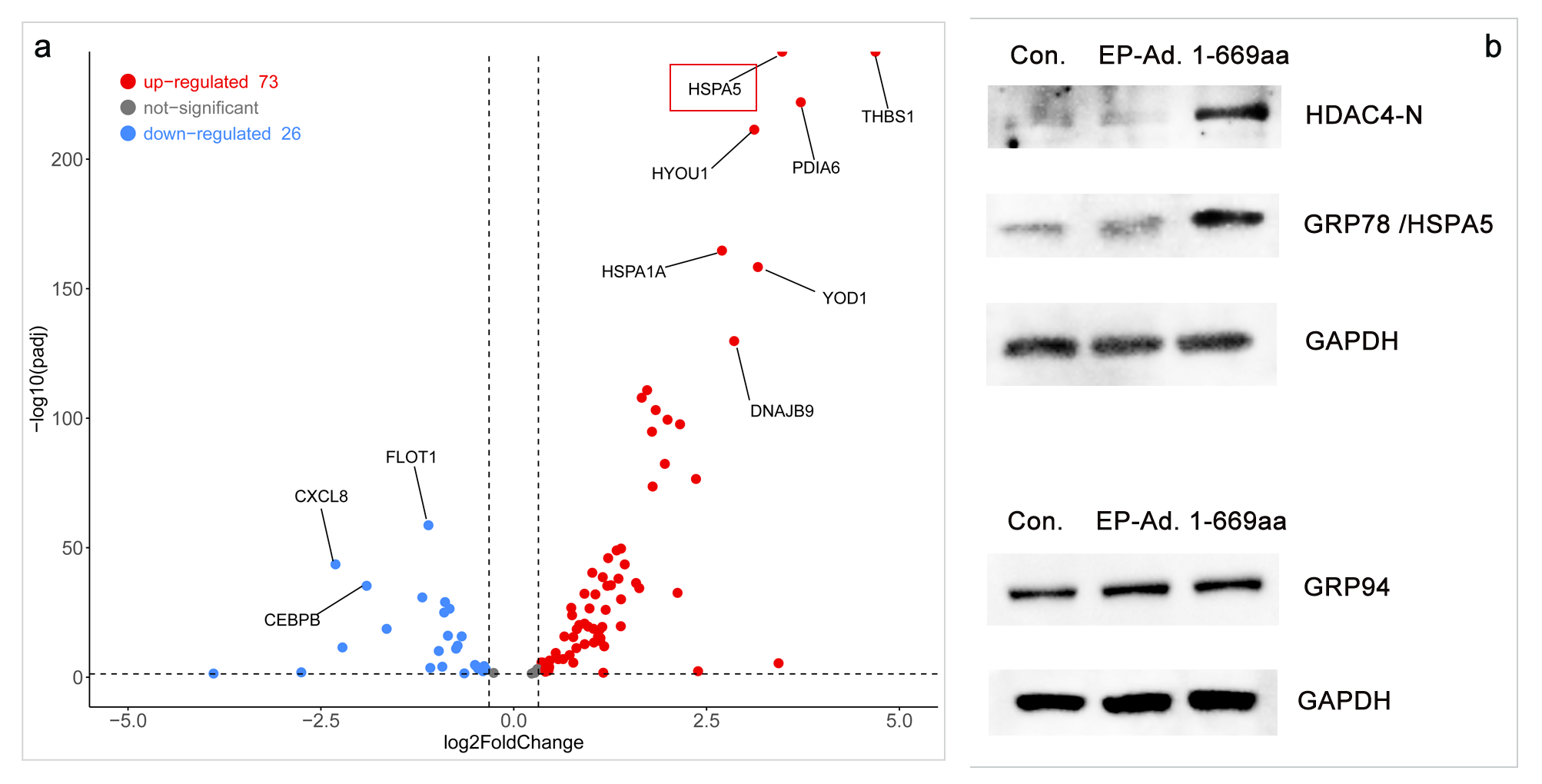

Supplement: Supplementary file 1 — Figure S1. [file JCMM-28-e70135-s002.zip › Figure-S-8 WB.png]

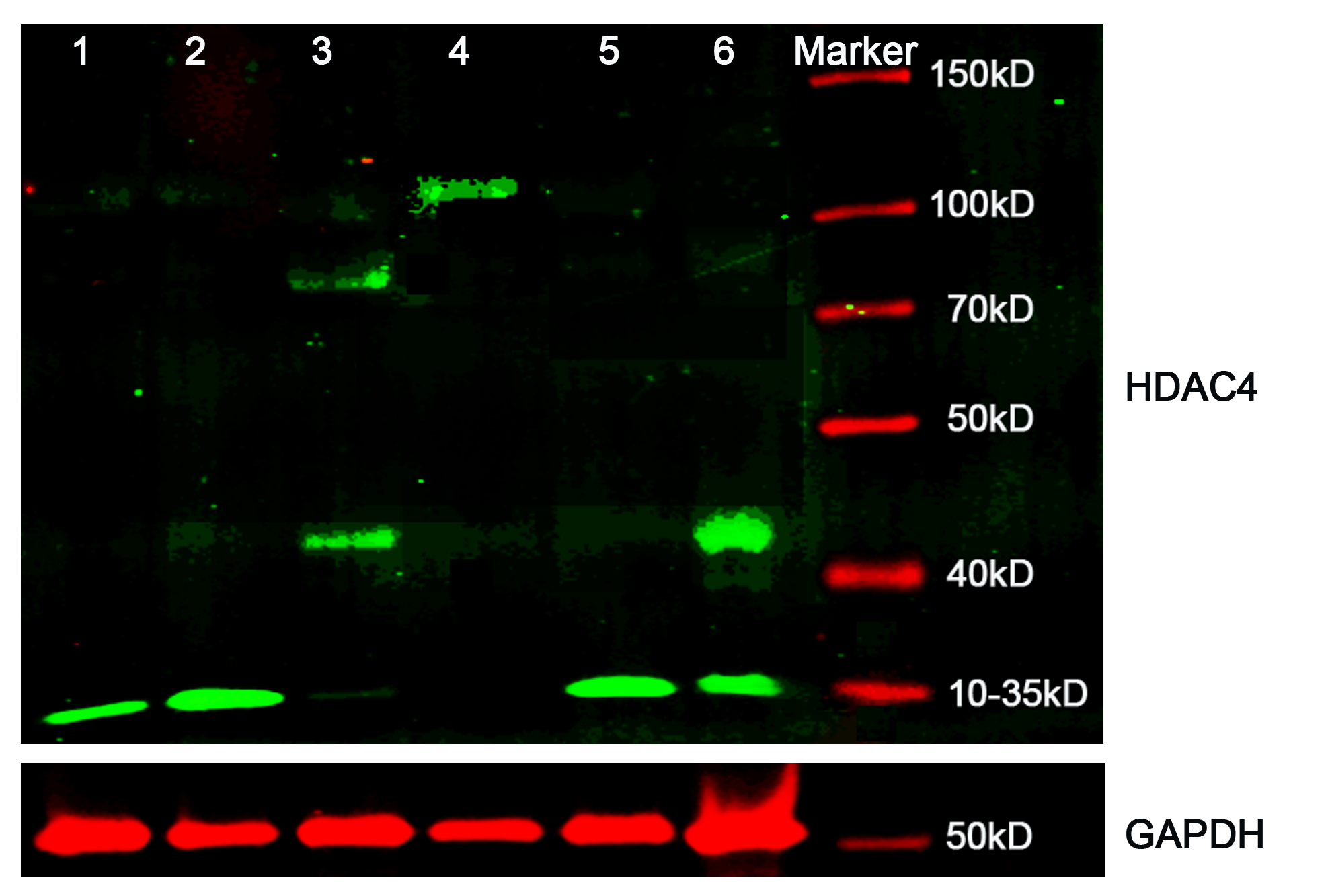

Supplement: Supplementary file 1 — Figure S1. [file JCMM-28-e70135-s002.zip › Figure-S-9.png]
