# Supplementary material for: The N‐terminal fragment of histone deacetylase 4 (1‐669aa) promotes chondrocyte apoptosis via the p53‐dependent endoplasmic reticulum stress pathway
Source: J Cell Mol Med. 2024 Oct 20;28(20):e70135. doi: 10.1111/jcmm.70135 (PMC11491302; doi:10.1111/jcmm.70135)
Supplement: Supplementary file 2 — Table S1. [file JCMM-28-e70135-s001.docx]

| **Table-1 Differentially expressed genes for H4_N(1-669aa) versus EP (top 20).** | | | | | | | | | | | | | | | | | | |  |
| --- | --- | --- | --- | --- | --- | --- | --- | --- | --- | --- | --- | --- | --- | --- | --- | --- | --- | --- | --- |
| ID | H4_N | | EP | | log2FoldChange | | | pvalue | padj | | gene_name | | gene strand | gene length | | gene_description | | |  |
| ENSG00000198938 | 3266.61 | | 73542.30 | | -4.49 | | | 0 | 0 | | MT-CO3 | | + | 784 | | mitochondrially encoded cytochrome c oxidase III [Source:HGNC Symbol;Acc:HGNC:7422] | | |  |
| ENSG00000198899 | 3702.21 | | 60368.18 | | -4.03 | | | 0 | 0 | | MT-ATP6 | | + | 681 | | mitochondrially encoded ATP synthase membrane subunit 6 [Source:HGNC Symbol;Acc:HGNC:7414] | | |  |
| ENSG00000198888 | 2383.83 | | 62217.35 | | -4.71 | | | 0 | 0 | | MT-ND1 | | + | 956 | | mitochondrially encoded NADH:ubiquinone oxidoreductase core subunit 1 [Source:HGNC Symbol;Acc:HGNC:7455] | | |  |
| ENSG00000044574 | 61593.91 | | 5516.51 | | 3.48 | | | 0 | 0 | | HSPA5 | | - | 3908 | | heat shock protein family A (Hsp70) member 5 [Source:HGNC Symbol;Acc:HGNC:5238] | | |  |
| ENSG00000198763 | 2774.65 | | 51292.97 | | -4.21 | | | 0 | 0 | | MT-ND2 | | + | 1042 | | mitochondrially encoded NADH:ubiquinone oxidoreductase core subunit 2 [Source:HGNC Symbol;Acc:HGNC:7456] | | |  |
| ENSG00000128591 | 21921.89 | | 3773.60 | | 2.54 | | | 0 | 0 | | FLNC | | + | 9280 | | filamin C [Source:HGNC Symbol;Acc:HGNC:3756] | | |  |
| ENSG00000128595 | 31227.75 | | 2726.85 | | 3.52 | | | 0 | 0 | | CALU | | + | 4537 | | calumenin [Source:HGNC Symbol;Acc:HGNC:1458] | | |  |
| ENSG00000137801 | 19131.44 | | 740.20 | | 4.69 | | | 0 | 0 | | THBS1 | | + | 9158 | | thrombospondin 1 [Source:HGNC Symbol;Acc:HGNC:11785] | | |  |
| ENSG00000068024 | 11408.07 | | 99.28 | | 6.85 | | | 0 | 0 | | HDAC4 | | - | 13368 | | histone deacetylase 4 [Source:HGNC Symbol;Acc:HGNC:14063] | | |  |
| ENSG00000187608 | 1619.19 | | 26023.57 | | -4.01 | | | 0 | 0 | | ISG15 | | + | 942 | | ISG15 ubiquitin-like modifier [Source:HGNC Symbol;Acc:HGNC:4053] | | |  |
| ENSG00000150938 | 11923.54 | | 1132.09 | | 3.40 | | | 0 | 0 | | CRIM1 | | + | 7122 | | cysteine rich transmembrane BMP regulator 1 [Source:HGNC Symbol;Acc:HGNC:2359] | | |  |
| ENSG00000116260 | 9829.29 | | 1812.22 | | 2.44 | | | 0 | 0 | | QSOX1 | | + | 9311 | | quiescin sulfhydryl oxidase 1 [Source:HGNC Symbol;Acc:HGNC:9756] | | |  |
| ENSG00000059804 | 10653.42 | | 1099.90 | | 3.27 | | | 0 | 0 | | SLC2A3 | | - | 6159 | | solute carrier family 2 member 3 [Source:HGNC Symbol;Acc:HGNC:11007] | | |  |
| ENSG00000119922 | 398.08 | | 6056.81 | | -3.93 | | | 0 | 0 | | IFIT2 | | + | 4101 | | interferon induced protein with tetratricopeptide repeats 2 [Source:HGNC Symbol;Acc:HGNC:5409] | | |  |
| ENSG00000091409 | 5140.70 | | 566.87 | | 3.18 | | | 0 | 0 | | ITGA6 | | + | 6533 | | integrin subunit alpha 6 [Source:HGNC Symbol;Acc:HGNC:6142] | | |  |
| ENSG00000145934 | 4746.52 | | 449.68 | | 3.40 | | | 0 | 0 | | TENM2 | | + | 11042 | | teneurin transmembrane protein 2 [Source:HGNC Symbol;Acc:HGNC:29943] | | |  |
| ENSG00000143507 | 7077.59 | | 808.82 | | 3.13 | | | 0 | 0 | | DUSP10 | | - | 2914 | | dual specificity phosphatase 10 [Source:HGNC Symbol;Acc:HGNC:3065] | | |  |
| ENSG00000085449 | 5581.58 | | 430.03 | | 3.70 | | | 0 | 0 | | WDFY1 | | - | 5516 | | WD repeat and FYVE domain containing 1 [Source:HGNC Symbol;Acc:HGNC:20451] | | |  |
| ENSG00000137193 | 6783.40 | | 730.40 | | 3.21 | | | 0 | 0 | | PIM1 | | + | 2977 | | Pim-1 proto-oncogene, serine/threonine kinase [Source:HGNC Symbol;Acc:HGNC:8986] | | |  |
| ENSG00000204388 | 6054.03 | | 209.43 | | 4.85 | | | 0 | 0 | | HSPA1B | | + | 2521 | | heat shock protein family A (Hsp70) member 1B [Source:HGNC Symbol;Acc:HGNC:5233] | | |  |
| EP, empty plasmid. H4_N, HDAC4_N terminal(1-669aa). p-adj, p-adjusted. | | | | | | | | | | | | | | | | | | |  |
| **Table-2 Differentially expressed genes for H4 versus EP (top 20).** | | | | | | | | | | | | | | | | | | |  |
| ID | | H4 | | EP | | log2FoldChange | pvalue | | | padj | | gene_name | | | gene strand | | gene length | gene_description | |
| ENSG00000137801 | | 11305.32 | | 762.19 | | 3.89 | 0 | | | 0 | | THBS1 | | | + | | 9158 | thrombospondin 1 [Source:HGNC Symbol;Acc:HGNC:11785] | |
| ENSG00000068024 | | 6750.69 | | 102.30 | | 6.05 | 0 | | | 0 | | HDAC4 | | | - | | 13368 | histone deacetylase 4 [Source:HGNC Symbol;Acc:HGNC:14063] | |
| ENSG00000167601 | | 11602.70 | | 1791.76 | | 2.69 | 0 | | | 0 | | AXL | | | + | | 5154 | AXL receptor tyrosine kinase [Source:HGNC Symbol;Acc:HGNC:905] | |
| ENSG00000150938 | | 11575.04 | | 1166.34 | | 3.31 | 0 | | | 0 | | CRIM1 | | | + | | 7122 | cysteine rich transmembrane BMP regulator 1 [Source:HGNC Symbol;Acc:HGNC:2359] | |
| ENSG00000131016 | | 5540.33 | | 827.71 | | 2.74 | 0 | | | 0 | | AKAP12 | | | + | | 8741 | A-kinase anchoring protein 12 [Source:HGNC Symbol;Acc:HGNC:370] | |
| ENSG00000128595 | | 18593.05 | | 2809.27 | | 2.73 | 3.101E-301 | | | 1.514E-297 | | CALU | | | + | | 4537 | calumenin [Source:HGNC Symbol;Acc:HGNC:1458] | |
| ENSG00000083857 | | 3184.66 | | 364.80 | | 3.13 | 6.295E-297 | | | 2.634E-293 | | FAT1 | | | - | | 16177 | FAT atypical cadherin 1 [Source:HGNC Symbol;Acc:HGNC:3595] | |
| ENSG00000145934 | | 3682.91 | | 463.25 | | 2.99 | 4.779E-291 | | | 1.75E-287 | | TENM2 | | | + | | 11042 | teneurin transmembrane protein 2 [Source:HGNC Symbol;Acc:HGNC:29943] | |
| ENSG00000131236 | | 4110.56 | | 482.50 | | 3.09 | 9.179E-283 | | | 2.988E-279 | | CAP1 | | | + | | 3814 | cyclase associated actin cytoskeleton regulatory protein 1 [Source:HGNC Symbol;Acc:HGNC:20040] | |
| ENSG00000150093 | | 34386.52 | | 6169.48 | | 2.48 | 2.406E-280 | | | 7.047E-277 | | ITGB1 | | | - | | 6011 | integrin subunit beta 1 [Source:HGNC Symbol;Acc:HGNC:6153] | |
| ENSG00000100292 | | 2661.39 | | 18654.76 | | -2.81 | 4.912E-273 | | | 1.308E-269 | | HMOX1 | | | + | | 2405 | heme oxygenase 1 [Source:HGNC Symbol;Acc:HGNC:5013] | |
| ENSG00000106366 | | 54598.62 | | 11535.19 | | 2.24 | 1.728E-271 | | | 4.219E-268 | | SERPINE1 | | | + | | 3190 | serpin family E member 1 [Source:HGNC Symbol;Acc:HGNC:8583] | |
| ENSG00000148677 | | 11736.02 | | 2326.49 | | 2.33 | 1.706E-256 | | | 3.845E-253 | | ANKRD1 | | | - | | 1979 | ankyrin repeat domain 1 [Source:HGNC Symbol;Acc:HGNC:15819] | |
| ENSG00000128591 | | 19001.79 | | 3887.62 | | 2.29 | 9.787E-249 | | | 2.048E-245 | | FLNC | | | + | | 9280 | filamin C [Source:HGNC Symbol;Acc:HGNC:3756] | |
| ENSG00000135245 | | 7715.98 | | 1546.66 | | 2.32 | 3.264E-248 | | | 6.375E-245 | | HILPDA | | | + | | 2070 | hypoxia inducible lipid droplet associated [Source:HGNC Symbol;Acc:HGNC:28859] | |
| ENSG00000196924 | | 52722.12 | | 12131.88 | | 2.12 | 7.46E-247 | | | 1.366E-243 | | FLNA | | | - | | 10585 | filamin A [Source:HGNC Symbol;Acc:HGNC:3754] | |
| ENSG00000134954 | | 10835.03 | | 2556.76 | | 2.08 | 2.51E-242 | | | 4.325E-239 | | ETS1 | | | - | | 7149 | ETS proto-oncogene 1, transcription factor [Source:HGNC Symbol;Acc:HGNC:3488] | |
| ENSG00000253729 | | 4698.14 | | 759.18 | | 2.63 | 5.89E-242 | | | 9.585E-239 | | PRKDC | | | - | | 15417 | protein kinase, DNA-activated, catalytic polypeptide [Source:HGNC Symbol;Acc:HGNC:9413] | |
| ENSG00000116260 | | 8619.06 | | 1867.08 | | 2.21 | 5.832E-240 | | | 8.991E-237 | | QSOX1 | | | + | | 9311 | quiescin sulfhydryl oxidase 1 [Source:HGNC Symbol;Acc:HGNC:9756] | |
| ENSG00000185164 | | 1919.17 | | 129.09 | | 3.89 | 3.129E-231 | | | 4.584E-228 | | NOMO2 | | | - | | 6801 | NODAL modulator 2 [Source:HGNC Symbol;Acc:HGNC:22652] | |
| EP, empty plasmid. H4, HDAC4. p-adj, p-adjusted.   \| **Table-3 Gene Ontology enrichment analysis for H4_N versus EP (top 10).** \| \| \| \| \| \| \| \| \| --- \| --- \| --- \| --- \| --- \| --- \| --- \| --- \| \| Category \| ID \| Description \| GeneRatio \| BgRatio \| pvalue \| padj \| Count \| \| BP \| GO:0000278 \| mitotic cell cycle \| 345/4533 \| 476/7691 \| 1.60E-10 \| 6.83E-07 \| 345 \| \| BP \| GO:0044265 \| cellular macromolecule catabolic process \| 357/4533 \| 496/7691 \| 3.16E-10 \| 6.83E-07 \| 357 \| \| BP \| GO:1903047 \| mitotic cell cycle process \| 293/4533 \| 409/7691 \| 2.95E-08 \| 4.19E-05 \| 293 \| \| BP \| GO:0007346 \| regulation of mitotic cell cycle \| 231/4533 \| 315/7691 \| 3.88E-08 \| 4.19E-05 \| 231 \| \| BP \| GO:0016071 \| mRNA metabolic process \| 283/4533 \| 398/7691 \| 1.61E-07 \| 0.000139 \| 283 \| \| BP \| GO:0061024 \| membrane organization \| 327/4533 \| 468/7691 \| 2.97E-07 \| 0.00018798 \| 327 \| \| BP \| GO:0097190 \| apoptotic signaling pathway \| 193/4533 \| 262/7691 \| 3.04E-07 \| 0.00018798 \| 193 \| \| BP \| GO:0010564 \| regulation of cell cycle process \| 249/4533 \| 348/7691 \| 3.98E-07 \| 0.00021489 \| 249 \| \| BP \| GO:0044257 \| cellular protein catabolic process \| 242/4533 \| 339/7691 \| 7.88E-07 \| 0.00033536 \| 242 \| \| BP \| GO:0009411 \| response to UV \| 55/4533 \| 63/7691 \| 8.44E-07 \| 0.00033536 \| 55 \| \| CC \| GO:1990234 \| transferase complex \| 273/4665 \| 365/8020 \| 8.94E-12 \| 4.86E-09 \| 273 \| \| CC \| GO:0030055 \| cell-substrate junction \| 162/4665 \| 207/8020 \| 5.63E-10 \| 1.53E-07 \| 162 \| \| CC \| GO:0005924 \| cell-substrate adherens junction \| 159/4665 \| 204/8020 \| 1.42E-09 \| 1.93E-07 \| 159 \| \| CC \| GO:0005925 \| focal adhesion \| 159/4665 \| 204/8020 \| 1.42E-09 \| 1.93E-07 \| 159 \| \| CC \| GO:0005912 \| adherens junction \| 193/4665 \| 255/8020 \| 2.13E-09 \| 2.31E-07 \| 193 \| \| CC \| GO:0070161 \| anchoring junction \| 199/4665 \| 266/8020 \| 6.33E-09 \| 5.40E-07 \| 199 \| \| CC \| GO:0005730 \| nucleolus \| 306/4665 \| 430/8020 \| 6.97E-09 \| 5.40E-07 \| 306 \| \| CC \| GO:0005813 \| centrosome \| 196/4665 \| 263/8020 \| 1.40E-08 \| 8.84E-07 \| 196 \| \| CC \| GO:0016604 \| nuclear body \| 255/4665 \| 353/8020 \| 1.47E-08 \| 8.84E-07 \| 255 \| \| CC \| GO:0005815 \| microtubule organizing center \| 236/4665 \| 331/8020 \| 3.07E-07 \| 1.67E-05 \| 236 \| \| MF \| GO:0044389 \| ubiquitin-like protein ligase binding \| 121/4587 \| 155/7734 \| 4.91E-07 \| 0.00036348 \| 121 \| \| MF \| GO:0031625 \| ubiquitin protein ligase binding \| 113/4587 \| 146/7734 \| 2.55E-06 \| 0.00094171 \| 113 \| \| MF \| GO:0019900 \| kinase binding \| 202/4587 \| 286/7734 \| 3.46E-05 \| 0.0085379 \| 202 \| \| MF \| GO:0003684 \| damaged DNA binding \| 31/4587 \| 35/7734 \| 0.000156 \| 0.02193525 \| 31 \| \| MF \| GO:0044877 \| macromolecular complex binding \| 321/4587 \| 478/7734 \| 0.000162 \| 0.02193525 \| 321 \| \| MF \| GO:0032403 \| protein complex binding \| 255/4587 \| 374/7734 \| 0.000178 \| 0.02193525 \| 255 \| \| MF \| GO:0004518 \| nuclease activity \| 115/4587 \| 158/7734 \| 0.000254 \| 0.02681971 \| 115 \| \| MF \| GO:0045296 \| cadherin binding \| 106/4587 \| 145/7734 \| 0.000333 \| 0.0297043 \| 106 \| \| MF \| GO:0008134 \| transcription factor binding \| 180/4587 \| 259/7734 \| 0.000361 \| 0.0297043 \| 180 \| \| MF \| GO:0019901 \| protein kinase binding \| 171/4587 \| 246/7734 \| 0.000495 \| 0.03379652 \| 171 \| \| BP, Biological Process. CC, Cell Component. MF,Molecular Function. \| \| \| \| \| \| \| \| | | | | | | | | | | | | | | | | | | | |

| **Table-4 Gene Ontology enrichment analysis for H4 versus EP (top 10).** | | | | | | | |
| --- | --- | --- | --- | --- | --- | --- | --- |
| Category | ID | Description | GeneRatio | BgRatio | pvalue | padj | Count |
| BP | GO:0044265 | cellular macromolecule catabolic process | 357/4182 | 497/7748 | 2.83E-17 | 1.23E-13 | 357 |
| BP | GO:0000278 | mitotic cell cycle | 340/4182 | 477/7748 | 1.13E-15 | 2.45E-12 | 340 |
| BP | GO:1903047 | mitotic cell cycle process | 296/4182 | 410/7748 | 6.24E-15 | 9.01E-12 | 296 |
| BP | GO:0007346 | regulation of mitotic cell cycle | 230/4182 | 316/7748 | 1.95E-12 | 2.11E-09 | 230 |
| BP | GO:0016071 | mRNA metabolic process | 278/4182 | 397/7748 | 1.52E-11 | 1.32E-08 | 278 |
| BP | GO:0032446 | protein modification by small protein conjugation | 306/4182 | 444/7748 | 2.81E-11 | 1.84E-08 | 306 |
| BP | GO:0044257 | cellular protein catabolic process | 243/4182 | 342/7748 | 2.98E-11 | 1.84E-08 | 243 |
| BP | GO:0016567 | protein ubiquitination | 284/4182 | 409/7748 | 4.13E-11 | 2.24E-08 | 284 |
| BP | GO:0044770 | cell cycle phase transition | 209/4182 | 290/7748 | 9.51E-11 | 4.58E-08 | 209 |
| BP | GO:0044772 | mitotic cell cycle phase transition | 201/4182 | 278/7748 | 1.38E-10 | 5.96E-08 | 201 |
| CC | GO:0030055 | cell-substrate junction | 159/4265 | 206/8083 | 2.00E-13 | 1.08E-10 | 159 |
| CC | GO:0005924 | cell-substrate adherens junction | 156/4265 | 203/8083 | 6.50E-13 | 1.17E-10 | 156 |
| CC | GO:0005925 | focal adhesion | 156/4265 | 203/8083 | 6.50E-13 | 1.17E-10 | 156 |
| CC | GO:0005912 | adherens junction | 188/4265 | 254/8083 | 1.38E-12 | 1.87E-10 | 188 |
| CC | GO:0070161 | anchoring junction | 193/4265 | 265/8083 | 8.56E-12 | 9.25E-10 | 193 |
| CC | GO:0098796 | membrane protein complex | 236/4265 | 335/8083 | 1.23E-11 | 1.11E-09 | 236 |
| CC | GO:0016604 | nuclear body | 245/4265 | 353/8083 | 5.81E-11 | 4.48E-09 | 245 |
| CC | GO:0005730 | nucleolus | 290/4265 | 430/8083 | 1.59E-10 | 1.07E-08 | 290 |
| CC | GO:0005694 | chromosome | 301/4265 | 450/8083 | 2.89E-10 | 1.74E-08 | 301 |
| CC | GO:1990234 | transferase complex | 247/4265 | 364/8083 | 1.59E-09 | 8.58E-08 | 247 |
| MF | GO:0045296 | cadherin binding | 111/4201 | 144/7788 | 5.56E-09 | 4.13E-06 | 111 |
| MF | GO:0044389 | ubiquitin-like protein ligase binding | 115/4201 | 155/7788 | 1.29E-07 | 4.81E-05 | 115 |
| MF | GO:0019904 | protein domain specific binding | 220/4201 | 327/7788 | 3.78E-07 | 9.37E-05 | 220 |
| MF | GO:0050839 | cell adhesion molecule binding | 158/4201 | 227/7788 | 7.47E-07 | 0.0001389 | 158 |
| MF | GO:0031625 | ubiquitin protein ligase binding | 107/4201 | 146/7788 | 9.92E-07 | 0.0001476 | 107 |
| MF | GO:0017111 | nucleoside-triphosphatase activity | 239/4201 | 363/7788 | 1.65E-06 | 0.0001953 | 239 |
| MF | GO:0019900 | kinase binding | 192/4201 | 285/7788 | 1.84E-06 | 0.0001953 | 192 |
| MF | GO:0004842 | ubiquitin-protein transferase activity | 145/4201 | 209/7788 | 2.85E-06 | 0.0002655 | 145 |
| MF | GO:0008134 | transcription factor binding | 175/4201 | 260/7788 | 5.73E-06 | 0.0004033 | 175 |
| MF | GO:0016462 | pyrophosphatase activity | 250/4201 | 386/7788 | 6.44E-06 | 0.0004033 | 250 |
| BP, Biological Process. CC, Cell Component. MF,Molecular Function. | | | | | | | |

| **Table-5 Kyoto Encyclopedia of Genes and Genomes pathway analysis for H4_N versus EP (top 20).** | | | | | | |
| --- | --- | --- | --- | --- | --- | --- |
| ID | Description | GeneRatio | BgRatio | pvalue | padj | Count |
| hsa05210 | Colorectal cancer | 62/3927 | 71/6388 | 1.27E-06 | 0.0003804 | 62 |
| hsa04141 | Protein processing in endoplasmic reticulum | 122/3927 | 155/6388 | 2.67E-06 | 0.0003804 | 122 |
| hsa04115 | p53 signaling pathway | 59/3927 | 68/6388 | 3.71E-06 | 0.0003804 | 59 |
| hsa05220 | Chronic myeloid leukemia | 65/3927 | 77/6388 | 9.05E-06 | 0.00069704 | 65 |
| hsa04110 | Cell cycle | 97/3927 | 122/6388 | 1.30E-05 | 0.0007337 | 97 |
| hsa04810 | Regulation of actin cytoskeleton | 151/3927 | 200/6388 | 1.43E-05 | 0.0007337 | 151 |
| hsa05211 | Renal cell carcinoma | 57/3927 | 67/6388 | 2.02E-05 | 0.00088771 | 57 |
| hsa04120 | Ubiquitin mediated proteolysis | 104/3927 | 133/6388 | 2.47E-05 | 0.00095102 | 104 |
| hsa05169 | Epstein-Barr virus infection | 131/3927 | 174/6388 | 6.65E-05 | 0.00227725 | 131 |
| hsa03040 | Spliceosome | 95/3927 | 122/6388 | 7.45E-05 | 0.00229534 | 95 |
| hsa04130 | SNARE interactions in vesicular transport | 31/3927 | 34/6388 | 0.000108561 | 0.00303971 | 31 |
| hsa04520 | Adherens junction | 58/3927 | 71/6388 | 0.000186764 | 0.00452411 | 58 |
| hsa05215 | Prostate cancer | 75/3927 | 95/6388 | 0.000192402 | 0.00452411 | 75 |
| hsa05212 | Pancreatic cancer | 60/3927 | 74/6388 | 0.000217654 | 0.00452411 | 60 |
| hsa04068 | FoxO signaling pathway | 95/3927 | 124/6388 | 0.000223274 | 0.00452411 | 95 |
| hsa00190 | Oxidative phosphorylation | 97/3927 | 127/6388 | 0.000235019 | 0.00452411 | 97 |
| hsa00562 | Inositol phosphate metabolism | 57/3927 | 70/6388 | 0.000251126 | 0.00454981 | 57 |
| hsa03013 | RNA transport | 120/3927 | 161/6388 | 0.000278034 | 0.00475748 | 120 |
| hsa01522 | Endocrine resistance | 71/3927 | 90/6388 | 0.000296566 | 0.0048075 | 71 |
| hsa00900 | Terpenoid backbone biosynthesis | 21/3927 | 22/6388 | 0.000326209 | 0.00493775 | 21 |

| **Table-6 Kyoto Encyclopedia of Genes and Genomes pathway analysis for H4 versus EP (top 20).** | | | | | | |
| --- | --- | --- | --- | --- | --- | --- |
| ID | Description | GeneRatio | BgRatio | pvalue | padj | Count |
| hsa04141 | Protein processing in endoplasmic reticulum | 121/3637 | 156/6448 | 1.92E-08 | 5.92E-06 | 121 |
| hsa00190 | Oxidative phosphorylation | 97/3637 | 127/6448 | 1.80E-06 | 0.0002767 | 97 |
| hsa03040 | Spliceosome | 92/3637 | 121/6448 | 4.67E-06 | 0.0003866 | 92 |
| hsa05166 | HTLV-I infection | 161/3637 | 228/6448 | 5.02E-06 | 0.0003866 | 161 |
| hsa05016 | Huntington's disease | 134/3637 | 187/6448 | 9.41E-06 | 0.0005796 | 134 |
| hsa03010 | Ribosome | 96/3637 | 129/6448 | 1.43E-05 | 0.0007348 | 96 |
| hsa04520 | Adherens junction | 57/3637 | 71/6448 | 1.88E-05 | 0.0008283 | 57 |
| hsa05215 | Prostate cancer | 73/3637 | 95/6448 | 2.38E-05 | 0.0009158 | 73 |
| hsa04120 | Ubiquitin mediated proteolysis | 99/3637 | 135/6448 | 3.00E-05 | 0.0010261 | 99 |
| hsa03013 | RNA transport | 114/3637 | 160/6448 | 6.52E-05 | 0.0020089 | 114 |
| hsa05210 | Colorectal cancer | 55/3637 | 71/6448 | 0.000165 | 0.0044897 | 55 |
| hsa05010 | Alzheimer's disease | 115/3637 | 164/6448 | 0.00018 | 0.0044897 | 115 |
| hsa04510 | Focal adhesion | 135/3637 | 196/6448 | 0.00019 | 0.0044897 | 135 |
| hsa01524 | Platinum drug resistance | 52/3637 | 67/6448 | 0.000228 | 0.0046873 | 52 |
| hsa05211 | Renal cell carcinoma | 52/3637 | 67/6448 | 0.000228 | 0.0046873 | 52 |
| hsa04066 | HIF-1 signaling pathway | 71/3637 | 96/6448 | 0.000258 | 0.0049648 | 71 |
| hsa01522 | Endocrine resistance | 67/3637 | 90/6448 | 0.000277 | 0.0050233 | 67 |
| hsa05161 | Hepatitis B | 93/3637 | 131/6448 | 0.000372 | 0.0063642 | 93 |
| hsa05222 | Small cell lung cancer | 67/3637 | 91/6448 | 0.000475 | 0.0077056 | 67 |
| hsa04810 | Regulation of actin cytoskeleton | 136/3637 | 201/6448 | 0.000603 | 0.0085551 | 136 |
